# Structural Modifications of Deoxycholic Acid to Obtain Three Known Brassinosteroid Analogues and Full NMR Spectroscopic Characterization

**DOI:** 10.3390/molecules21091139

**Published:** 2016-08-27

**Authors:** Heidy Herrera, Rodrigo Carvajal, Andrés F. Olea, Luis Espinoza

**Affiliations:** 1 Departamento de Química, Universidad Técnica Federico Santa María, Av. España No. 1680, Valparaíso 2340000, Chile; heidy.herrera@postgrado.usm.cl (H.H.); rodrigo.carvajal@postgrado.usm.cl (R.C.); 2 Instituto de Ciencias Químicas Aplicadas, Facultad de Ingeniería, Universidad Autónoma de Chile, Santiago 8910339, Chile; andres.olea@uautonoma.cl

**Keywords:** synthesis, deoxycholic acid derivatives, brassinosteroid analogues, full NMR spectroscopic characterization

## Abstract

An improved synthesis route for obtaining known brassinosteroid analogues, i.e., methyl 2α,3α-dihydroxy-6-oxo-5α-cholan-24-oate (**11**), methyl 3α-hydroxy-6-oxo-7-oxa-5α-cholan-24-oate (**15**) and methyl 3α-hydroxy-6-oxa-7-oxo-5α-cholan-24-oate (**16**), from hyodeoxycholic acid (**4**) maintaining the native side chain is described. In the alternative procedure, the di-oxidized product **6**, obtained in the oxidation of methyl hyodeoxycholate **5**, was converted almost quantitatively into the target monoketone **7** by stereoselective reduction with NaBH_4_, increasing the overall yield of this synthetic route to 96.8%. The complete ^1^H- and ^13^C-NMR assignments for all compounds synthesized in this work have been made by 1D and 2D heteronuclear correlation *gs*-HSQC and *gs*-HMBC techniques. Thus, it was possible to update the spectroscopic information of ^1^H-NMR and to accomplish a complete assignment of all ^13^C-NMR signals for analogues **5**–**16**, which were previously reported only in partial form.

## 1. Introduction

**Figure 1:**
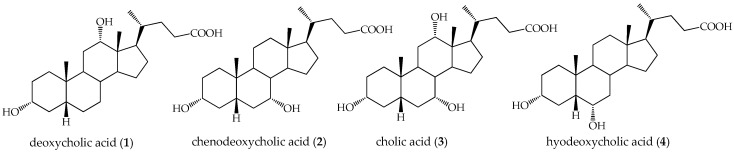
Structure of bile acids used for the synthesis of brassinolide and its analogues.

Bile acids, such as deoxycholic (**1**), chenodeoxycholic (**2**), cholic (**3**) and hyodeoxycholic (**4**) (Figure 1), have been used as substrates for the synthesis of a large number of brassinosteroid analogues, keeping the methyl ester or carboxylic function in the side chain [1,2,3,4,5,6,7]. In particular, hyodeoxycholic acid (**4**) has been used because it contains the modifiable organic functions at suitable positions, satisfying the structural requirements on the A and/or B and A/B *trans* fusion ring and side chain of active brassinosteroids [8,9,10,11,12,13,14,15,16,17,18,19,20,21,22,23,24,25,26]. Hyodeoxycholic acid (HDA) extracted from hog bile was initially used as a precursor for steroid synthesis [27]. It has also been shown that HDA prevents cholesterol-induced gallstones in animals fed with a lithogenic diet [28], whereas oral administration of HDA leads to a decrease in the LDL-cholesterol concentration, a strong stimulation of hepatic cholesterol biosynthesis and an excessive loss of cholesterol in feces [29].

In this work, we report an improved synthesis route to obtain known brassinosteroid analogues, starting from hyodeoxycholic acid (**4**), and maintaining the native side chain. Additionally, the full NMR spectroscopic characterization of these derivatives is performed.

## 2. Results and Discussion

**Scheme 1:**
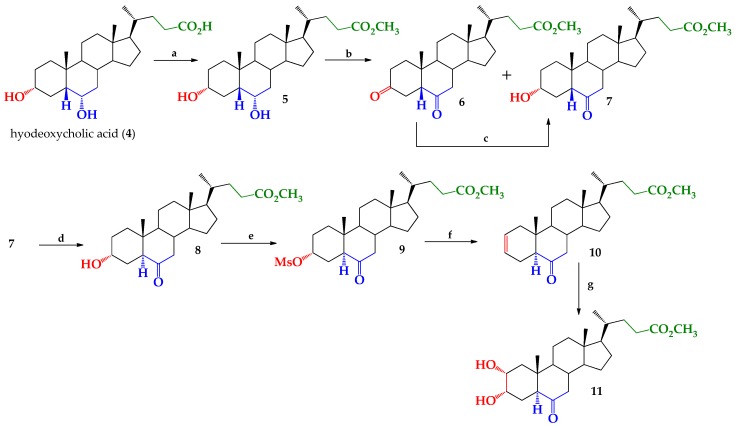
Synthesis of hyodeoxycholic acid derivatives **5**–**10** and brassinosteroid analogue **11**. *Reagents and conditions*: (**a**) CH_3_OH/H_2_SO_4_, reflux, 2 h; (**b**) PCC/DCM, 48 h., r.t., C.C. separation; (**c**) NaBH_4_/MeOH, 0–5 °C, 1 h; (**d**) HCl/CH_3_OH 2.5%, r.t., 24 h; (**e**) CH_3_SO_2_Cl/DCM, DMAP, 0 °C ≥ r.t. 1 h; (**f**) Li_2_CO_3_/LiBr, DMF, 80 °C, 1.5 h; (**g**) OsO_4_ 4%/NMO, acetone, r.t., 72 h.

Fischer esterification of hyodeoxycholic acid (**4**) with the H_2_SO_4_-MeOH system gave the methyl hyodeoxycholate (**5**) in almost quantitative yield (98%). Subsequently, selective oxidation of the C-6 hydroxyl group with PCC/CH_2_Cl_2_ afforded the di- and mono-oxidized compounds **6** and **7** with 30% and 68% yield, respectively. These yields are similar to those reported when PDC/CH_2_Cl_2_ was the oxidizing agent [13]. However, the yield of compound **7** was 67%–70% when K_2_CrO_4_ was used as an oxidizing agent [30,31]. Thus, in order to increase the yield of this reaction, **6** was conveniently converted to the desired monoketone **7** by selective reduction with NaBH_4_/MeOH at a low temperature (0–5 °C) with 96.8% yield (Scheme 1). This reaction has been used to modify steroids with similar structures [24]. The overall yield of **7** is increased from 68% to 96.8%. The physical properties and spectroscopic data (IR and ^1^H-NMR) of compounds **6** and **7** were consistent with those previously reported [8,30,31,32]. The ^13^C-NMR spectroscopic data for compound **7** has been informed but not assigned [32].

**Figure 2:**
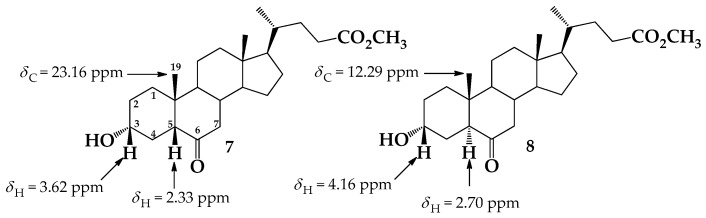
Major differences observed in the ^1^H- and ^13^C-NMR data of compounds **7** and **8**, associated with the change from the 5β-cholestan to 5α-cholestan skeleton (A/B ring *cis* for *trans* fusion).

Compound **7** was readily isomerized (94.4% yield) under acid conditions (2.5% HCl/MeOH) to give the derivative **8** possessing 5α-cholestan-6-one skeleton [8,13,32]. This isomerization reaction can be performed under alkaline conditions as well, but under these conditions the obtained compound was isomerized and hydrolyzed (carboxylic acid) with 84% yield [31]. Thus, to get **8**, an additional esterification reaction was carried out with 94% yield. The physical properties, IR, and ^1^H-NMR spectroscopic data of compound **8** have been previously reported, but the signals of ^13^C-NMR were not assigned [32]. The change of the 5β-cholestan to 5α-cholestan skeleton (A/B ring *cis* for *trans* fusion) implies a very important structural modification, and therefore a detailed analysis of NMR spectroscopic data for compounds **7** and **8** must be performed. The main differences found in the ^1^H- and ^13^C-NMR data are shown in Figure 2. The signals corresponding to H-3β and H-5α (δ_H_ = 4.16 and δ_H_ = 2.70 ppm, respectively) in compound **8**, are displaced to downfield as compared to compound **7** (δ_H_ = 3.62 and δ_H-5β_ = 2.33 ppm, respectively). On the other hand, the signal of CH_3_-19 in the ^13^C-NMR of compound **8** appears at a higher field (δ_C_ = 12.29 ppm) than in the spectrum of compound **7** (δ_C_ = 23.16 ppm).

**Scheme 2:**
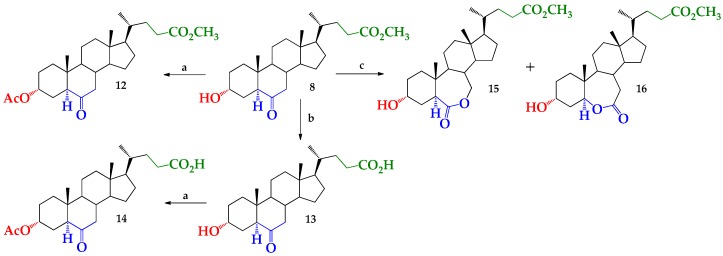
Synthesis of hyodeoxycholic acid derivatives **8**, **12**–**14** and known 7-oxalactone **15** and 6-oxalactone **16**. *Reagents and conditions*: (**a**) Ac_2_O/DMAP, CH_2_Cl_2_/py, r.t., 30 min; (**b**) K_2_CO_3_/CH_3_OH, reflux, 1 h; (**c**) *m*-CPBA/CH_2_Cl_2_, NaHCO_3_ r.t., 24 h.

The reaction of compound **8** with methanesulfonyl chloride (mesyl chloride) in CH_2_Cl_2_/DMAP produces the mesylated derivative **9** with 61% yield [28]. The major ^1^H-NMR spectroscopic evidence for formation of this derivative is the presence of signals at δ_H_ = 5.02 and 2.97 ppm assigned to hydrogen H-3β (b.s. 1H) and CH_3_-SO_2_- (s, 3H), respectively, whereas in the ^13^C-NMR spectrum, a signal corresponding to CH_3_-SO_2_ was observed at δ_C_ = 38.38 ppm. Subsequent treatment of compound **9** with the Li_2_CO_3_/LiBr system at 80 °C in dimethylformamide (DMF) produces the alquene **10** with 74.9% yield [33] (Scheme 2). The ^1^H-NMR spectroscopic data of alquene **10** was consistent with that previously reported [8,32]. Additionally, in the ^13^C-NMR spectrum the signals at δ_C_ = 124.49 (C-2) and 124.95 (C-3) ppm are observed. Both signals were assigned from the 2D HMBC spectrum, where the H-3 (δ_H_ = 5.69–5.66 ppm, m, 1H) showed ^3^*J*_H-C_ correlation with C-1 (δ_C_ = 39.34 ppm) and ^2^*J*_H-C_ with C-4 (δ_C_ = 27.88 ppm), while H-2 (δ_H_ = 5.57–5.55 ppm, m, 1H) showed ^3^*J*_H-C_ correlation with C-4 (δ_C_ = 27.88 ppm) and ^2^*J*_H-C_ with C-1 (δ_C_ = 39.34 ppm).

Compound **11** was obtained with 51% yield by stereospecific α-face hydroxylation of olefin **10** using a catalytic amount of osmium tetraoxide (4.0%) in a mixture of (CH_3_)_2_CO-H_2_O (3:1) and 2.0 mL of pyridine, and in the presence of *N*-methylmorpholine *N*-oxide [33]. The ^1^H- and ^13^C-NMR spectroscopic data of diol **11** were consistent with those reported [8,32]. In addition, the spatial orientations of α-OH in C-2 and α-OH in C-3 were determined by selective-excitation 1D NOESY experiments, where H-2β (3.77 ppm, b.d, *J* = 11.1 Hz, 1H) showed a long-range spatial correlation with CH_3_-19 (0.75 ppm, s, 3H), while the observed signals at δ_C_ = 68.29 and 68.39 ppm in the ^13^C-NMR spectrum were assigned to C-2 and C-3, respectively.

In order to get additional deoxycholic acid derivatives, which may be of interest, the chemical transformations described in Scheme 2 were performed. Thus, compound **8** was acetylated under standard conditions (Ac_2_O/DMAP/CH_2_Cl_2_), and commercial compound **12** was obtained with 95% yield. The synthesis of this derivative has been previously reported, but no NMR spectroscopic data was included [25]. The ^1^H-NMR spectrum of the acetylated derivative **12** shows a singlet at δ_H_ = 2.04 ppm (3H, CH_3_CO) and a multiplet at δ_H_ = 5.12 ppm corresponding to H-3β (m, 1H). The latter is shifted to downfield as compared with H-3β (δ_H_ = 4.16 ppm, m 1H) in compound **8**. Additionally, in the ^13^C-NMR spectrum the signals appearing at δ_C_ = 170.31 (C=O) and 21.39 (CH_3_CO) ppm (Table 1) confirmed the presence of acetylated derivative **12**.

The synthesis of compound **13** by saponification of **8** with NaOH/dioxane under reflux conditions has been previously informed [31]. Saponification of compound **8** under mild conditions (K_2_CO_3_/MeOH, reflux) produces the carboxylic acid **13** with similar yields (82.2% yield) (Scheme 2). The physical property data, IR and ^1^H-NMR were consistent with those reported [31]. The ^13^C-NMR spectrum shows a signal at δ_C_ = 178.19 ppm (C=O) (Table 1), confirming the presence of the carboxylic function. Standard acetylation (Ac_2_O/DMAP/CH_2_Cl_2_) of **13** produces compound **14** with 72.8% yield. Compound **14** showed signals at 3373–2495 (OH), 1736 (C=O) and 1708 (C=O) cm^−1^ in the IR spectrum, while in ^1^H-NMR the signals observed at δ_H_ = 5.12 ppm (m, 1H) and 2.03 ppm (s, 3H) were assigned to the H-3β and CH_3_CO, respectively. In the ^13^C-NMR spectrum the observed signals at δ_C_ = 170.33 and 21.41 ppm (Table 1) were assigned to the acetyl group, while the signal at δ_C_ = 179.20 ppm (Table 1) was assigned to the carboxylic function. These spectroscopic data confirmed the structure of compound **14**.

The Baeyer-Villiger oxidation of 3α-hydroxy-6-oxo-5α-cholanate **8** with *m*-CPBA/CH_2_Cl_2_ gave a mixture of known 7-oxalactone **15** and 6-oxalactone **16** with 10.8% and 14.9% yields, respectively. The physical and IR, ^1^H-NMR spectroscopic data of **15** and **16** were consistent with those reported for these compounds [14,25]. Considering that the original ^1^H-NMR spectra were recorded with a 60 MHz spectrometer, and no ^13^C-NMR data was reported, we believe that it is worth updating the analysis of NMR data for these compounds.

**Figure 3:**
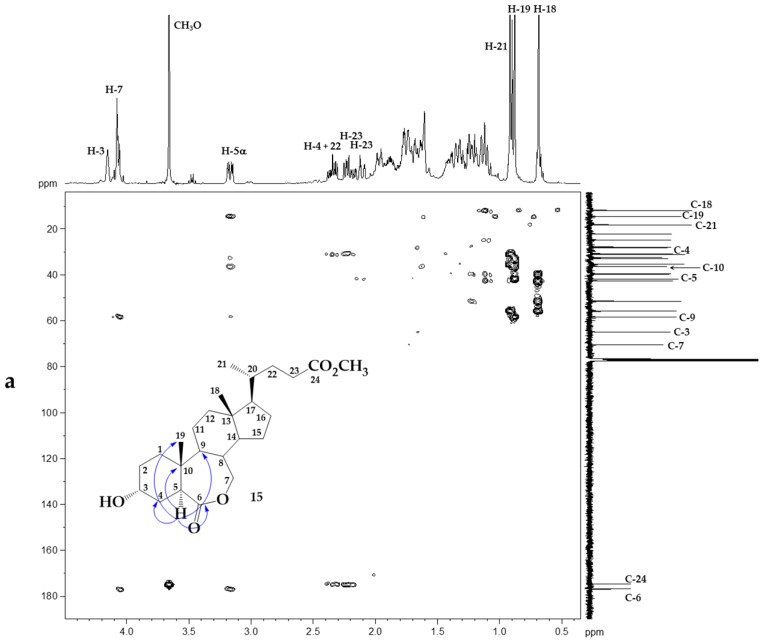

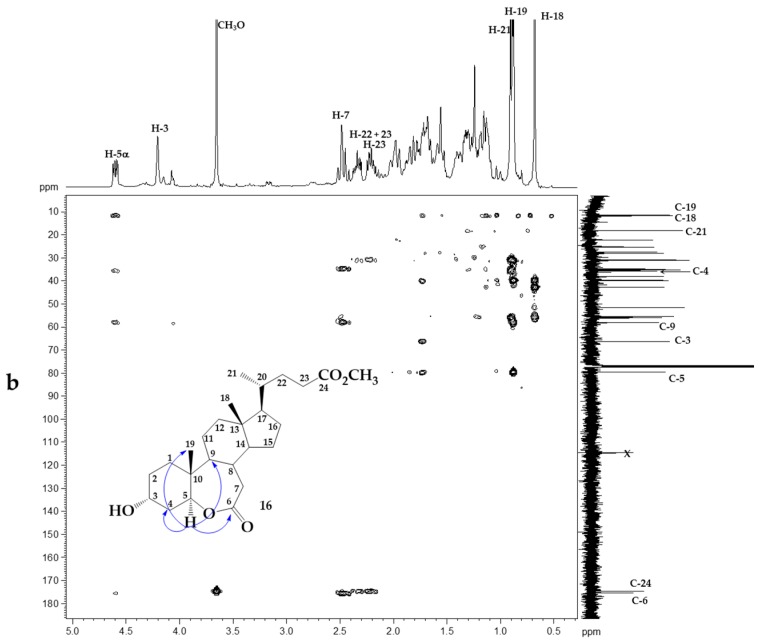
(**a**) Inverse detection heteronuclear-correlated 2D ^1^H-^13^C HMBC contour plot and major observed ^2^*J*_HC_ and ^3^*J*_HC_ correlations for H-5α in 7-oxalactone **15** and (**b**) for 6-oxolactone **16**.

The full structural assignment of compound **15** and in particular the 7-oxalactone position was mainly assigned by ^1^H, ^13^C, 2D HSQC and 2D HMBC NMR spectroscopy. In the ^1^H-NMR spectrum of compound **15** appears a signal at δ_H_ = 4.08–4.06 ppm (m, 2H), which was assigned to the two hydrogens H-7 and correlated by 2D ^1^H-^13^C HSQC with the signal δ_C_ = 70.44 ppm (C-7) with pair multiplicity (from DEPT-135 analysis). Additionally, from the 2D ^1^H-^13^C HMBC spectrum important heteronuclear correlations were observed, i.e., ^2^*J*_HC_ correlations between H-5α (δ_H_ = 3.17 ppm, dd, *J* = 4.4 and 12.2 Hz) with the signals at δ_C_ = 176.76 (C=O of lactone function), δ_C_ = 36.29 and δ_C_ = 30.81 ppm which were assigned to the carbons C-6, quaternary C-10 and C-4, respectively. Additionally, H-5α showed ^3^*J*_HC_ correlation with signals at δ_C_ = 30.81, δ_C_ = 14.52, δ_C_ = 32.88 and δ_C_ = 58.35 ppm, which were assigned to the carbons C-4, CH_3_-19, C-1 and C-9, respectively (Figure 3a). These 2D HMBC observations unequivocally confirm the 7-oxalactone position for compound **15**.

A similar analysis was performed for the structure determination of 6-oxolactone **16**; thus, in the ^1^H-NMR spectrum for **16** a signal at δ_H_ = 4.60 ppm (dd, 1H, *J* = 5.3 and 11.3 Hz) was observed, correlated by 2D ^1^H-^13^C HSQC with the signal at δ_C_ = 79.65 ppm (C-7) with impair multiplicity (from DEPT-135 analysis) and assigned to H-5α. Additionally, H-5 α showed ^2^*J*_HC_ correlation with signals at δ_C_ = 35.63 ppm and ^3^*J*_HC_ correlation with signals at δ_C_ = 11.53, δ_C_ = 57.97 and δ_C_ = 175.35 ppm, which were assigned to the carbons C-4, CH_3_-19, C-9 and C-6 (C=O, of lactone function), respectively (Figure 3b).

## 3. Materials and Methods

### 3.1. General

All reagents were purchased from commercial suppliers (Merck, Darmstadt, Germany or Aldrich, St. Louis, MO, USA), and used without further purification. Melting points were measured on a Stuart-Scientific SMP3 apparatus (Staffordshire, UK) and are uncorrected. ^1^H, ^13^C, ^13^C DEPT-135, sel. gs1D ^1^H NOESY, gs 2D HSQC and gs 2D HMBC NMR spectra were recorded in CDCl_3_ or MeOD solutions, and are referenced to the residual peaks of CHCl_3_ at δ = 7.26 ppm and δ = 77.00 ppm for ^1^H and ^13^C, respectively and CD_3_OD at δ = 3.30 ppm and δ = 49.00 ppm for ^1^H and ^13^C, respectively, on a Bruker Avance 400 Digital NMR spectrometer (Bruker, Rheinstetten, Germany), operating at 400.1 MHz for ^1^H and 100.6 MHz for ^13^C. Chemical shifts are reported in δ ppm and coupling constants (*J*) are given in Hz. IR spectra were recorded as KBr disks in a FT-IR Nicolet 6700 spectrometer (Thermo Scientific, San Jose, CA, USA) and frequencies are reported in cm^−1^. For analytical TLC, silica gel 60 (Merck) in 0.25 mm layer was used and TLC spots were detected by heating after spraying with 25% H_2_SO_4_ in H_2_O. Chromatographic separations were carried out by conventional column on silica gel 60 (230–400 mesh, Merck) using EtOAc-hexane gradients of increasing polarity. All organic extracts were dried over anhydrous magnesium sulfate and evaporated under reduced pressure, below 40 °C.

### 3.2. Synthesis

*Methyl 3α, 6α-dihydroxy-5β-cholan-24-oate* (**5**). A solution of **4** (20.0 g, 50.95 mmol) in MeOH (150 mL) and 1.0 mL of H_2_SO_4_ was refluxed for 2.0 h. The end of reaction was verified by TLC. Then the solvent was removed (until a 40 mL approximate volume) and diluted with EtOAc (90 mL). The organic layer was washed with saturated solution of NaHCO_3_ (40 mL) and water (2 × 30 mL), dried over Na_2_SO_4_, and filtered. The solvent was evaporated under reduced pressure. The crude was re-dissolved in CH_2_Cl_2_ (15 mL) and chromatographed on silica gel with EtOAc/hexane mixtures of increasing polarity (0.2:9.8 → 7.6:2.4). Compound **5** (19.50 g 98% yield) was a colorless solid (m.p. = 60–65 °C, MeOH/Et_2_O); IR (cm^−1^): 3385 (O-H); 2938 (C-CH_3_); 2858 (C-CH_2_-C); 1743 (C=O); 1452 (CH_2_); 1376 (CH_3_); 1169 (C-O). ^1^H-NMR: 4.05 (ddd, *J* = 4.8, 4.8 and 11.9 Hz, 1 H, H-6); 3.66 (s, 3H, CH_3_O); 3.62 (m, 1H, H-3); 2.35 (ddd, *J* = 5.1, 10.1 and 15.0 Hz, 1H, H-23); 2.21 (ddd, *J* = 6.6, 9.6 and 15 Hz, 1H, H-23); 0.91 (d, *J* = 5.1 Hz, 3H, H-21); 0.90 (s, 3H, H-19); 0.63 (s, 3H, H-18). ^13^C-NMR: See Table 2.

*Methyl 3, 6-dioxo-5β-cholan-24-oate* (**6**) and *Methyl 3α-hydroxy-6-oxo-5β-cholan-24-oate* (**7**). To a solution of **5** (16.50 g, 40.58 mmol) in DCM (100 mL), 8.75 g, (40.58 mmol) of PCC in 60 mL of DCM, were added by slow dripping. The reaction mixture was slowly stirred for 48 h at room temperature and the end of reaction was verified by TLC, filtered on silica (pore size 60 Å, 220–440 mesh) with DCM 30 mL. The solvent was evaporated under reduced pressure and the crude was re-dissolved in CH_2_Cl_2_ (5 mL) and chromatographed on silica gel with EtOAc/hexane mixtures of increasing polarity (0.2:9.8 → 5.8:4.2). Two fractions were obtained: Fraction I, 4.95 g (30.0% yield) of compound **6**; Fraction II, 11.2 g (68% yield) of compound **7**. Compound **6** was a colorless solid (m.p. = 106–107 °C, MeOH/Et_2_O). IR (cm^−1^): 2947 (C-CH_3_); 2874 (C-CH_3_); 1743 (C=O); 1717 (C=O); 1689 (C=O); 1469 (CH_2_); 1383 (CH_3_); 1164 (C-O). ^1^H-NMR: 3.64 (s, 3H, CH_3_O); 2.63 (dd, *J* = 13.4 and 14.7 Hz, 1H, H-12); 0.93 (s, 3H, H-19); 0.91 (d, *J* = 6.5 Hz, 3H, H-21); 0.67 (s, 3H, H-18). ^13^C-NMR: See Table 2. Compound **7** was a colorless solid (m.p. = 95–100 °C, MeOH/Et_2_O). IR (cm^−1^): 3301 (O-H); 2943 (C-CH_3_); 2867 (C-CH_2_-C); 2849 (C-CH_2_-C); 2828 (CH_3_O); 1740 (C=O); 1709 (C=O); 1436 (CH_2_); 1326 (CH_3_); 1171 (C-O). ^1^H-NMR: 3.66 (s, 3H, CH_3_O); 3.62 (m, 1H, H-3); 2.33 (ddd, *J* = 5, 10 and 15.2 Hz, 1H, H-23); 0.92 (d, *J* = 6.4 Hz, 3H, H-21); 0.83 (s, 3H, H-19); 0.64 (s, 3H, H-18). ^13^C-NMR: See Table 2. ^1^H-NMR and ^13^C-NMR are shown in Supplementary Materials.

*Methyl 3α-hydroxy-6-oxo-5β-cholan-24-oate* (***7***). A solution of compound **6** (4.95 g, 12.3 mmol) was prepared in 100 mL of MeOH. This solution was placed in a bath of ice-water between 0–5 °C. Subsequently 0.47 g (12.3 mmol) of NaBH_4_ were added in four portions (approximately 0.118 g each) maintaining the temperature and with slow stirring. The end of reaction was verified by TLC, so 20 mL of acetone and 5 mL of HCl 2.5% were added, maintaining the reaction temperature. The reaction mixture was concentrated by evaporation under reduced pressure to a volume of about 15 mL, and then AcOEt (50 mL) was added. The organic layer was washed with saturated solution of NaHCO_3_ (20 mL) and water (2 × 30 mL), dried over Na_2_SO_4_, and filtered. The solvent was evaporated under reduced pressure. The crude was re-dissolved in CH_2_Cl_2_ (5 mL) and chromatographed on silica gel with EtOAc/hexane mixtures of increasing polarity (0.2:9.8 → 5.8:4.2) Two fractions were obtained: Fraction I, 2.88 g of unreacted compound **6**; Fraction II, 2.04 g (41.2%) of compound **7**. Later reduction with NaHB_4_/CH_3_OH at 0–5 °C of recovered compound **6** (2.88 g), produced 2.75 g of **7** with 95.5% yield. The total yield in both reductions for compound **7** was 96.8%. The physical and spectroscopic properties of compound **7** were identical to those reported above for oxidation of compound **5**. ^1^H-NMR and ^13^C-NMR are shown in Supplementary Materials.

*Methyl 3α-hydroxy-6-oxo-5α-cholan-24-oate* (**8**). Compound **7** (4.79 g, 11.8 mmol) was dissolved in 100 mL of 2.5% *v/v* HCl-MeOH, at room temperature and constant agitation for 24 h. The end of reaction was verified by TLC. The solvent was evaporated under reduced pressure and the crude was re-dissolved in 40 mL of AcOEt. The organic layer was washed with saturated solution of NaHCO_3_ (30 mL) and water (2 × 30 mL), dried over Na_2_SO_4_, and filtered. The solvent was evaporated under reduced pressure. The crude was re-dissolved in CH_2_Cl_2_ (5 mL) and chromatographed on silica gel with EtOAc/hexane mixtures of increasing polarity (0.2:9.8 → 6.0:4.0). Compound **8** (4.79 g, 94.4% yield) was a colorless solid (m.p. = 128–132 °C, MeOH/Et_2_O) IR (cm^−1^): 3302 (O-H); 2943 (C-CH_3_); 2867 (C-CH_2_-C); 2849 (C-CH_2_-C); 2828 (CH_3_O); 1740 (C=O); 1709 (C=O); 1436 (CH_2_); 1375 (CH_3_); 1257 (C-O); 1172 (C-O). ^1^H-NMR: 4.16 (m, 1H, H-3); 3.66 (s, 3H, CH_3_O); 2.70 (t, *J* = 7.9 Hz, 1H, H-5); 2.33 (ddd, *J* = 5.3, 10.3 and 15.5 Hz, 1H, H-23); 0.91 (d, *J* = 6.4 Hz, 3H, H-21); 0.72 (s, 3H, H-19); 0.65 (s, 3H, H-18). ^13^C-NMR: See Table 2. ^1^H-NMR and ^13^C-NMR are shown in Supplementary Materials.

*Methyl 3α-methanesulfonyl-6-oxo-5α-cholan-24-oate* (**9**). Compound **8** (1.00 g, 2.47 mmol) was dissolved in 20 mL of DCM, 5 mg of DMAP and 1 mL of pyridine were added, then the mixture was cooled to 0–5 °C. Later 1.0 mL (12.9 mmol) of CH_3_SO_2_Cl slowly was added with gentle agitation. The mixture was kept at 0 °C for 30 min and subsequently left at room temperature. The end of reaction was verified by TLC. The solvent was evaporated under reduced pressure and the crude was re-dissolved in 20 mL of AcOEt. The organic layer was washed with saturated solution of NaHCO_3_ (40 mL) and water (2 × 30 mL), dried over Na_2_SO_4_, and filtered. The solvent was evaporated under reduced pressure. The crude was re-dissolved in CH_2_Cl_2_ (5 mL) and chromatographed on silica gel with EtOAc/hexane mixtures of increasing polarity (0.2:9.8 → 5.8:4.2). Two fractions were obtained: Fraction I, 726 mg (61.0% yield) of compound **9**, and Fraction II, 230 mg of unreacted compound **8**. Compound **9** was a colorless solid (m.p. = 129–131 °C, MeOH/Et_2_O) IR (cm^−1^): 2945 (C-CH_3_); 2867 (C-CH_2_-C); 1736 (C=O); 1709 (C=O); 1435 (CH_2_); 1351 (CH_3_); 1255 (C-O); 1171 (C-O). ^1^H-NMR: 5.02 (b.s, 1H, H-3); 3.64 (s, 3H, CH_3_O); 2.97 (s, 3H, CH_3_SO_3_); 2.60 (dd, *J* = 2.3 and 12.4 Hz, 1H, H-5); 0.901 (d, *J* = 6.4 Hz, 3H, H-21); 0.713 (s, 3H, H-19); 0.639 (s, 3H, H-18). ^13^C-NMR: See Table 2. ^1^H-NMR and ^13^C-NMR are shown in Supplementary Materials.

*Methyl 2-en-6-oxo-5α-cholan-24-oate* (**10**). To a solution of compound **9** (1.30 g, 0.269 mmol) in 40 mL of DMF, 200 mg Li_2_CO_3_ (2.71 mmol) and LiBr (235 mg, 2.71 mmol) were added. Then the reaction mixture was stirred and refluxed at 80 °C for 1.5 h. The end of reaction was verified by TLC, the mixture was then filtered and the mother liquor concentrated to a volume of approximately 15 mL under reduced pressure. Then AcOEt (30 mL) were added. The organic layer was washed with water (2 × 20 mL), dried over Na_2_SO_4_, and filtered. The solvent was evaporated under reduced pressure and the crude was re-dissolved in CH_2_Cl_2_ (5 mL) and chromatographed on silica gel with EtOAc/hexane mixtures of increasing polarity (0.2:9.8 → 2.4:7.6). Compound **10** (0.900 g, 74.9% yield) was a colorless solid (m.p. = 67–69 °C, hexane/Et_2_O) IR (cm^−1^): 3020 (CH=); 2968 (CH_3_-); 2939 (CH_3_-); 2899 (C-CH_2_-C); 2867 (C-CH_2_-C); 1737 (C=O); 1703 (C=O); 1635 (C=C); 1435 (CH_2_); 1383 (CH_3_); 1250, (C-O); 1168 (C-O). ^1^H-NMR: 5.69–5.66 (m, 1H, H-3); 5.57–5.55 (m, 1H, H-2); 3.66 (s, 3H, CH_3_O); 0.924 (d, *J* = 6.4 Hz, 3H, H-21); 0.701 (s, 3H, H-19); 0.665 (s, 3H, H-18). ^13^C-NMR: See Table 2. ^1^H-NMR and ^13^C-NMR are shown in Supplementary Materials.

*Methyl 2α,3α-dihydroxy-6-oxo-5α-cholan-24-oate* (**11**). A solution of compound **10** (500 mg, 1.29 mmol) in 30 mL of acetone and 10 mL water was prepared. Later 10 mg of NMMNO, 2 mL 4.0% (0,314 mmol) OsO_4_ solution and 2 mL of pyridine were added. The mixture was kept under constant stirring at room temperature for 72 h. The end of reaction was verified by TLC, and then 25 mL of Na_2_S_2_O_3_ saturated solution were added and allowed to stir for 30 min. The reaction was concentrated to a volume of approximately 15 mL under reduced pressure. Then AcOEt (40 mL) were added. The organic layer was washed with water (2 × 25 mL), dried over anhydrous Na_2_SO_4_, and filtered. The solvent was evaporated under reduced pressure and the crude was re-dissolved in CH_2_Cl_2_ (5 mL) and chromatographed on silica gel with EtOAc/hexane mixtures of increasing polarity (0.2:9.8 → 8.2:1.8). Two fractions were obtained: Fraction I, 218 mg of unreacted compound **10** and Fraction II, 277 mg (51.0% yield) of compound **11**. Compound **11** was a colorless solid (m.p. = 178–179 °C, MeOH/Et_2_O). IR (cm^−1^): 3392 (OH); 2944 (CH_3_-); 2867 (C-CH_2_-C); 1738 (C=O); 1709 (C=O); 1435 (CH_2_); 1376 (CH_3_); 1255, C-O); 1170 (C-O). ^1^H-NMR: 4.05 (b.s, 1H, H-3); 3.77 (b.d, *J* = 11.1 Hz, 1H, H-2); 3.66 (s, 3H, CH_3_O); 2.67 (dd, *J* = 3.0 and 12.6 Hz, 1H, H-5); 0.926 (d, *J* = 6.4 Hz, 3H, H-21); 0.752 (s, 3H, H-19); 0.659 (s, 3H, H-18). ^13^C-NMR: See Table 2. ^1^H-NMR and ^13^C-NMR are shown in Supplementary Materials.

*Methyl 3α-acetoxy-6-oxo-5α-cholan-24-oate* (**12**). To a solution of compound **8** (2.00g, 4.94 mmol) in 20 mL of DCM, 5 mg of DMAP, 1 mL of pyridine and 0.5 mL (5.3 mmol) of Ac_2_O were added. The reaction mixture was kept under constant stirring and room temperature for 30 min. The end of reaction was verified by TLC, the mixture was then concentrated to a volume approximately 5 mL under reduced pressure. Then AcOEt (30 mL) were added. The organic layer was washed with water (2 × 15 mL), dried over Na_2_SO_4_, and filtered. The solvent was evaporated under reduced pressure and the crude was re-dissolved in CH_2_Cl_2_ (5 mL) and chromatographed on silica gel with EtOAc/hexane mixtures of increasing polarity (0.2:9.8 → 4.7:5.3). Compound **12** (1.9 g, 95% yield) was a colorless solid (m.p. = 174–176 °C, MeOH/Et_2_O) IR (cm^−1^): 2943 (CH_3_); 2869 (CH_2_); 1737 (C=O); 1708 (C=O); 1435 (CH_2_); 1376 (CH_3_); 1262 (C-O); 1221 (C-O); 1172 (C-O). ^1^H-NMR: 5.12 (m, 1H, H-3); 3.66 (s, 3H, CH_3_O); 2.56 (dd, *J* = 3.3 and 12.0 Hz, 1H, H-5); 2.38 (ddd, *J* = 5.4, 10.3 and 15.4 Hz, 1H, H-23); 2.04 (s, 3H, CH_3_CO); 0.94 (d, *J* = 6.4 Hz, 3H, H-21); 0.74 (s, 3H, H-19); 0.67 (s, 3H, H-18). ^13^C-NMR: See Table 1. ^1^H-NMR and ^13^C-NMR are shown in Supplementary Materials.

*Acid 3α-hydroxy-6-oxo-5α-cholan-24-oic* (**13**). A solution of compound **8** (5.0 g, 12.36 mmol) in 60 mL of MeOH was prepared. Later 20 mL of 15% *P/V* K_2_CO_3_ solution were added, and the reaction mixture was refluxed for 1 h. The end of reaction was verified by TLC, the mixture was then concentrated to a volume approximately 15 mL under reduced pressure. This was acidified with 20 mL of 10% HCl solution. Then AcOEt (40 mL) were added. The organic layer was washed with water (2 × 15 mL), dried over Na_2_SO_4_, and filtered. The solvent was evaporated under reduced pressure and a colorless solid (4.11 g, 82.2% yield) was obtained. Compound **13** (m.p. = 102–107 °C, MeOH/Et_2_O) IR (cm^−1^): 3403–2500 (O-H); 2945 (CH_3_); 2869 (CH_2_); 1712 (C=O); 1689 (C=O); 1442 (CH_2_); 1380 (CH_3_). ^1^H-NMR (CD_3_OD): 4.04 (m, 1H, H-C3); 2.75 (t, *J* = 8.1 Hz, 1H, H-5); 2.31 (ddd, *J* = 5.3, 9.9 and 15.2 Hz, 1H, H-23); 0.96 (d, *J* = 6.5 Hz, 3H, H-21); 0.730 (s, 3H, H-19); 0.71 (s, 3H, H-18). ^13^C-NMR: See Table 1. ^1^H-NMR and ^13^C-NMR are shown in Supplementary Materials.

*Acid 3α-acetoxy-6-oxo-5α-cholan-24-oic* (**14**). To a solution of compound **13** (5.0 g, 12.8 mmol) in 60 mL of DCM, 10 mg of DMAP, 1 mL of pyridine and 1.2 mL (12.8 mmol) of Ac_2_O were added. The reaction mixture was kept under constant stirring and room temperature for 1 h. The end of reaction was verified by TLC, the mixture was then concentrated to a volume approximately 10 mL under reduced pressure. Then AcOEt (40 mL) were added. The organic layer was washed with 5% HCl (1 × 10 mL), water (2 × 15 mL), dried over Na_2_SO_4_, and filtered. The solvent was evaporated under reduced pressure and the crude was re-dissolved in CH_2_Cl_2_ (5 mL) and chromatographed on silica gel with EtOAc/hexane mixtures of increasing polarity (0.2:9.8 → 8.8:11.2). Compound **14** (3.64 g, 72.8% yield) was a colorless solid (m.p. = 171–177 °C, MeOH/Et_2_O) IR (cm^−1^): 3373-2495 (O-H); 2947 (CH_3_); 2869 (CH_2_); 1736 (C=O); 1708 (C=O); 1444 (CH_2_); 1376 (CH_3_). ^1^H-NMR: 5.12 (m, 1H, H-3); 2.56 (dd, *J* = 3.3 and 12.0 Hz, 1H, H-5); 2.38 (ddd, *J* = 5.4, 10.3 and 15.4 Hz, 1H, H-23); 2.03 (s, 3H, CH_3_CO); 0.94 (d, *J* = 6.4 Hz, 3H, H-21); 0.74 (s, 3H, H-19); 0,67 (s, 3H, H-18). ^13^C-NMR: See Table 1. ^1^H-NMR and ^13^C-NMR are shown in Supplementary Materials.

*Methyl 3α-hydroxy-6-oxo-7-oxa-5α-cholan-24-oate* (**15**) and *methyl 3α-hydroxy-6-oxa-7-oxo-5α-cholan-24-oate* (**16**). To a solution of **8** (100 mg, 0.247 mmol) in 60 mL of DCM, 55.3 mg (0.320 mmol) of *m*-CPBA (77%) and 269 mg (0.320 mmol) of NaHCO_3_ were added and the mixture was stirred at room temperature by 24 h. The end of reaction was verified by TLC, the mixture was filtered then concentrated to a volume approximately 5 mL under reduced pressure. Then AcOEt (40 mL) were added. The organic layer was washed with saturated NaHCO_3_ solution (2 × 20 mL), water (2 × 15 mL), dried over Na_2_SO_4_, and filtered. The solvent was evaporated under reduced pressure and the crude was re-dissolved in CH_2_Cl_2_ (5 mL) and chromatographed on silica gel with EtOAc/hexane mixtures of increasing polarity (0.2:9.8 → 6.0:4.0) Three fractions were obtained. Fraction I: 37 mg of unreacted compound **8**. Fraction II: 11.2 mg (10.8% yield) of compound **15** and Fraction III: 15.5 mg (14.9% yield) of compound **16**. Compound **15** was a colorless solid (m.p. = 140–142 °C, MeOH/Et_2_O) IR (cm^−1^): 3446 (O-H); 2947 (C-CH_3_); 2871 (C-CH_2_-C); 2849 (C-CH_2_-C); 1732 (C=O); 1436 (CH_2_); 1373 (CH_3_); 1251 (C-O); 1168 (C-O). ^1^H-NMR: 4.15 (b.s. 1H, H-3); 4.08-4.06 (m, 2H, H-7); 3.66 (s, 3H, CH_3_O); 3.17 (dd, *J* = 4.4 and 12.2 Hz, 1H, H-5); 0.907 (d, *J* = 6.5 Hz, 3H, H-21); 0.878 (s, 3H, H-19); 0.700 (s, 3H, H-18). ^13^C-NMR: See Table 1. Compound **16** was a colorless solid (m.p. = 155–157 °C). IR (cm^−1^): 3446 (O-H); 2946 (C-CH_3_); 1732 (C=O); 1444 (CH_2_); 1377 (CH_3_); 1250 (C-O); 1166 (C-O). ^1^H-NMR: 4.60 (dd, *J* = 5.3 and 11.3 Hz, 1H, H-5); 4.20 (b.s. 1H, H-3); 3.65 (s, 3H, CH_3_O); 0.897 (d, *J* = 6.6 Hz, 3H, H-21); 0.878 (s, 3H, H-19); 0.678 (s, 3H, H-18). ^13^C-NMR: See Table 1. ^1^H-NMR and ^13^C-NMR are shown in Supplementary Materials

## 4. Conclusions

An improved synthesis of known brassinosteroid analogues 7-oxalactone **15**, 6-oxalactone **16** and the 2α,3α-diol **11** from hyodeoxycholic acid has been described (**4**). These compounds and intermediates were obtained through modification of hyodeoxycholic acid as described previously. In the alternative procedure described here, the co-product **6** obtained in the oxidation of methyl hyodeoxycholate **5** was converted into the target monoketone **7** by stereoselective reduction with NaBH_4_, increasing the overall yield of this synthetic route to 96.8%.

Additionally, using mono- and bi-dimensional NMR techniques, we have updated the ^1^H-NMR spectroscopic information for derivatives of hyodeoxycholic acid **5**–**16**, and a complete assignment of all ^13^C-NMR signals has also been made.

Derivatives **8**, **13** and **14** will be used in the synthesis of new brassinosteroid analogues, while derivatives **8**, **11**, **15** and **16** will be evaluated as plant-growth regulators in the Rice Lamina Inclination Assay and for antifungal activity in plants. These results will be reported later.

## Figures and Tables

**Table 1:** Chemical shifts, δ, of ^13^C-NMR (CDCl_3_, 100.6 MHz) for compounds **12**–**16**.

C	12	13 *	14	15	16
1	32.38	32.87	32.36	32.88	27.46
2	27.89	28.96	27.88	22.13	30.75
3	68.87	66.01	68.85	64.88	66.31
4	25.26	28.70	25.28	30.81	35.63
5	52.58	52.83	52.56	41.78	79.65
6	211.86	215.65	211.84	176.76	175.35
7	46.71	47.59	46.73	70.44	38.07
8	37.91	39.41	37.93	39.45	35.27
9	53.73	55.03	53.72	58.35	57.97
10	41.26	43.71	41.24	36.29	39.85
11	21.06	22.17	21.09	24.80	22.17
12	39.44	40.78	39.47	39.68	39.66
13	43.02	44.16	43.07	42.66	42.71
14	55.79	57.18	55.76	55.72	56.00
15	25.00	28.42	25.00	28.23	25.24
16	23.88	24.92	23.87	27.82	27.93
17	56.74	57.89	56.75	51.45	55.48
18	12.01	12.41	12.01	11.79	11.78
19	12.39	12.68	12.40	14.51	11.53
20	35.24	36.60	35.25	35.31	34.83
21	18.19	18.72	18.17	18.14	18.12
22	30.67	32.01	30.65	31.01	30.99
23	30.84	32.24	30.83	32.49	31.15
24	174.67	178.19	179.20	174.60	174.64
CH_3_O	51.45	-	-	51.50	51.52
CH_3_CO-	170.31	-	170.33	-	-
CH_3_CO-	21.39	-	21.41	-	-

* The ^13^C-NMR spectrum of compound **13** was recorded in CD_3_OD solution.

**Table 2:** Chemical shifts, δ, of ^13^C-RMN (CDCl_3_, 100.6 MHz) for compounds **5**–**11**.

C	5	6	7	8	9	10	11
**1**	35.56	35.71	34.37	31.65	27.80	39.34	40.18
**2**	29.20	36.42	34.86	28.16	31.64	124.49	68.29
**3**	71.48	208.58	70.15	65.41	78.71	124.95	68.39
**4**	34.86	39.83	29.85	27.90	26.56	27.88	26.28
**5**	48.40	59.67	59.40	51.65	51.78	53.35	50.69
**6**	67.98	210.75	213.88	212.70	211.08	211.96	214.15
**7**	30.11	42.08	37.97	46.83	46.52	46.95	46.73
**8**	34.79	36.61	37.06	37.94	37.79	37.67	35.65
**9**	39.81	40.83	39.60	53.77	53.35	53.82	53.66
**10**	35.90	38.22	39.99	41.53	40.99	40.00	42.98
**11**	20.72	21.23	20.83	21.03	20.94	21.08	21.17
**12**	39.92	39.41	42.91	39.45	39.25	39.44	39.35
**13**	42.80	43.02	43.09	42.89	42.90	42.84	42.55
**14**	55.89	55.74	55.79	55.71	56.58	56.69	56.61
**15**	28.10	27.88	27.98	27.67	51.79	53.35	23.91
**16**	24.17	23.85	23.96	23.88	23.79	21.70	27.91
**17**	56.14	56.70	56.82	56.73	55.62	55.75	55.69
**18**	11.98	11.92	11.96	12.00	11.92	11.92	12.01
**19**	23.48	22.40	23.16	12.29	12.39	13.48	13.55
**20**	35.32	35.19	35.28	35.29	35.19	35.29	35.30
**21**	18.21	18.18	18.23	18.21	18.13	18.23	18.22
**22**	30.91	30.80	30.90	30.89	30.79	30.90	31.04
**23**	31.02	30.95	31.05	31.03	30.94	31.03	30.89
**24**	174.71	174.50	174.67	174.67	174.56	174.64	174.72
CH_3_O-	51.48	51.44	51.52	51.50	51.42	51.49	51.54
MsO-	-	-	-	-	38.38	-	-

## Supplementary Materials

Supplementary materials can be accessed at: http://www.mdpi.com/1420-3049/21/9/1139/s1.

## Abbreviations

The following abbreviations are used in this manuscript:

TLA: Three letter acronym
LD: linear dichroism
DCM: Dichloromethane
PCC: Pyridinium ChloroChromate
PDC: Pyridinium Dichromate
DMAP: 4-Dimethylaminopyridine
DMF: Dimethylformamide
NMO: *N*-Methylmorpholine-N-Oxide
*m*-CPBA: 3-Chloro or *metha*-chloroperoxybenzoic acid
NOESY: Nuclear Overhauser SpectroscopY
DEPT-135: Distortionless Enhancement by Polarization Transfer with flip angle of 135°
gs: gradient selected
HSQC: Heteronuclear Single Quantum Coherence
HMBC: Heteronuclear Multiple Bond Correlation
