# Supplementary material for: Structural Modifications of Deoxycholic Acid to Obtain Three Known Brassinosteroid Analogues and Full NMR Spectroscopic Characterization
Source: Molecules. 2016 Aug 27;21(9):1139. doi: 10.3390/molecules21091139 (PMC6274158; doi:10.3390/molecules21091139)
Supplement: Supplementary file 1 [file molecules-21-01139-s001.pdf]

Supplementary Materials: Structural Modifications of Deoxycholic Acid to Obtain Three Known Brassinosteroid Analogues and Full NMR Spectroscopic Characterization

Heidy Herrera, Rodrigo Carvajal, Andrés F. Olea and Luis Espinoza

NMR Spectra

Methyl 3,6-dioxo-5β-cholan-24-oate (**6**)

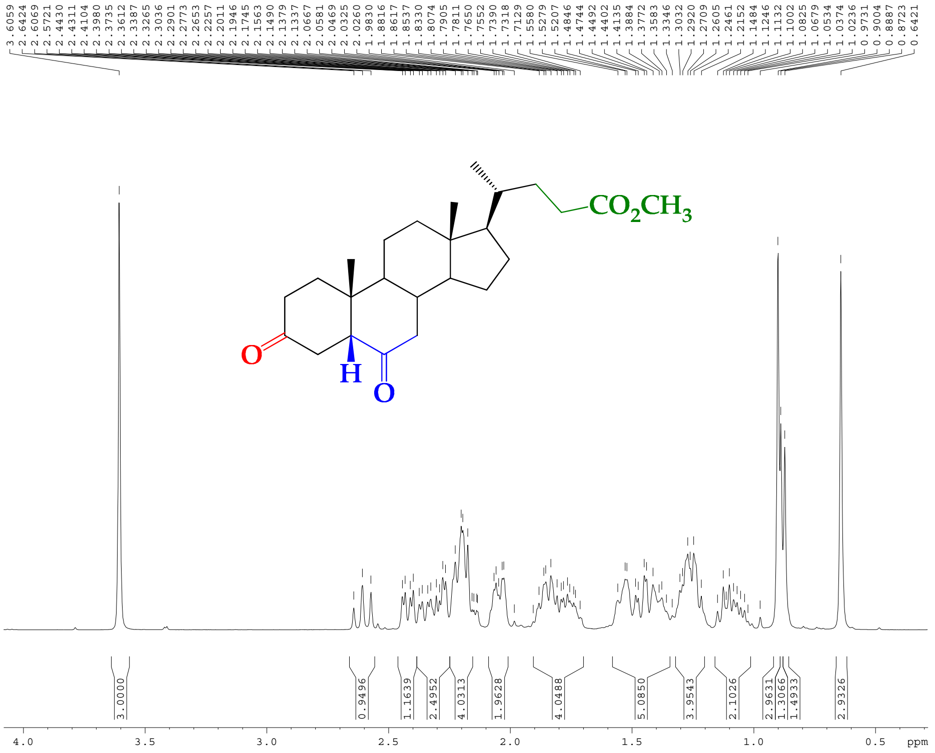

Figure S1. ^{1}H-NMR of Methyl 3,6-dioxo-5β-cholan-24-oate (**6**).

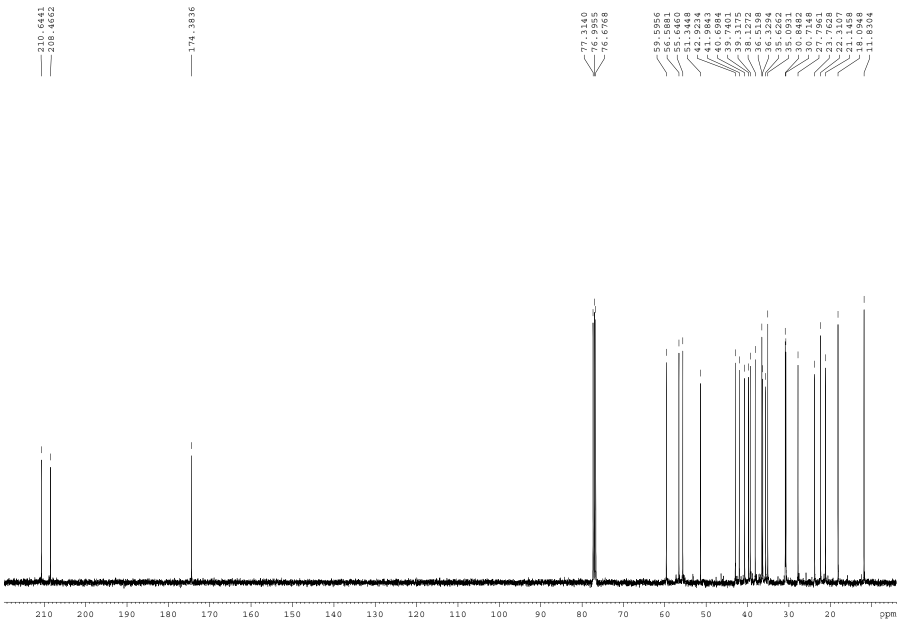

Figure S2. ^{13}C-NMR of Methyl 3,6-dioxo-5β-cholan-24-oate (**6**).

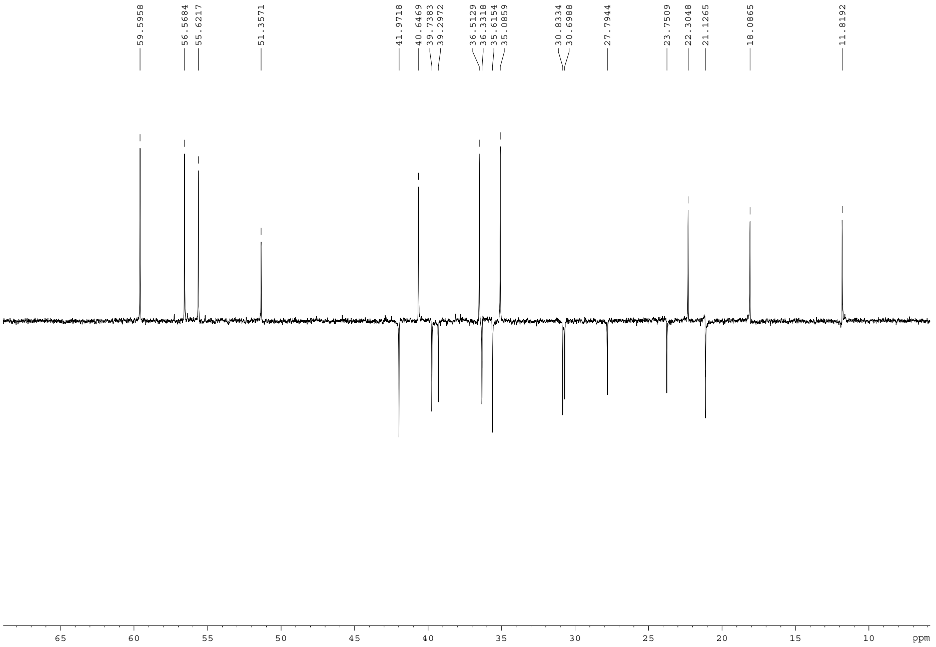

Figure S3. ^{13}C DEPT-135 NMR of Methyl 3,6-dioxo-5β-cholan-24-oate (**6**).

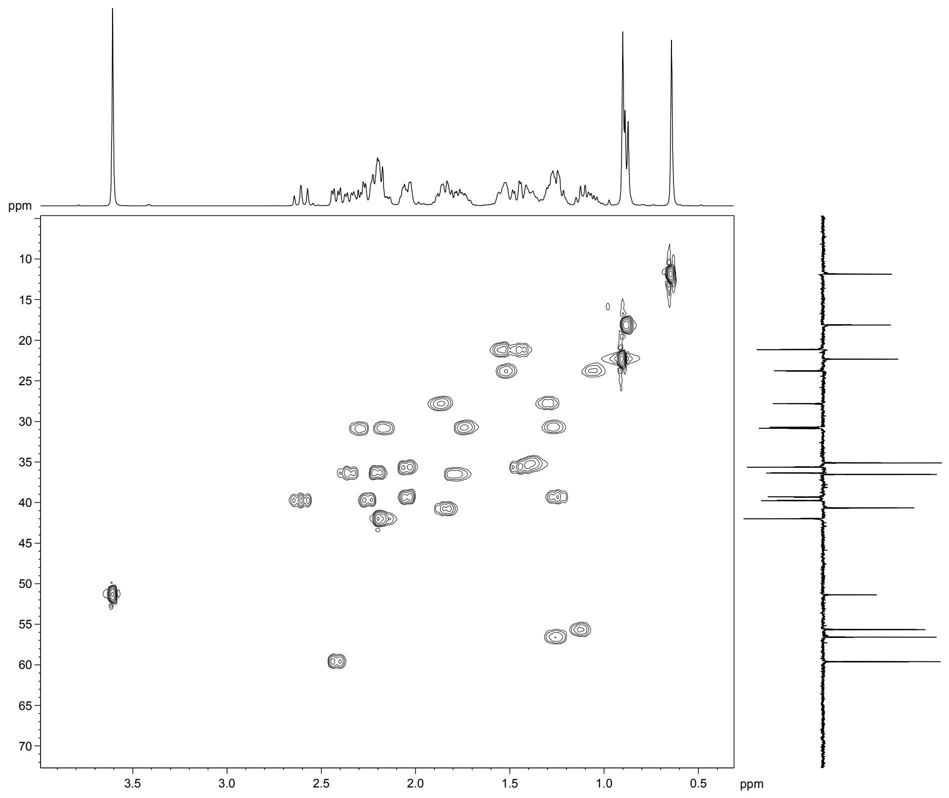

Figure S4. ^{1}H-^{13}C 2D HSQC of Methyl 3,6-dioxo-5β-cholan-24-oate (**6**).

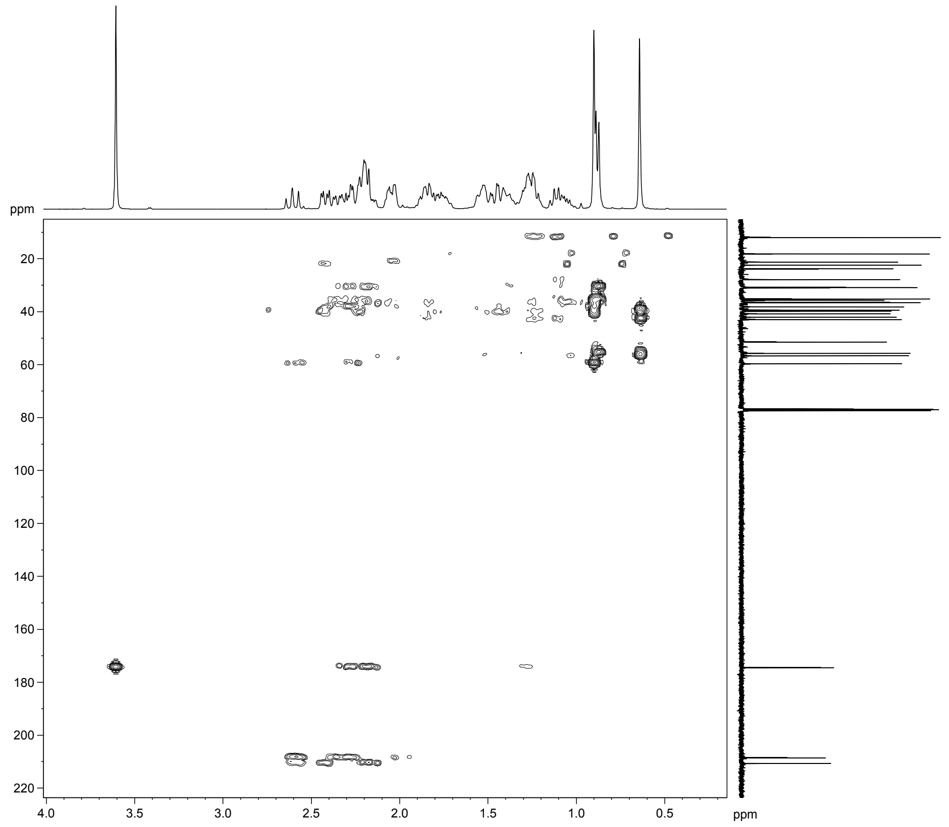

Figure S5. ^{1}H-^{13}C 2D HMBC of Methyl 3,6-dioxo-5β-cholan-24-oate (**6**).

Methyl 3α-hydroxy-6-oxo-5β-cholan-24-oate (**7**)

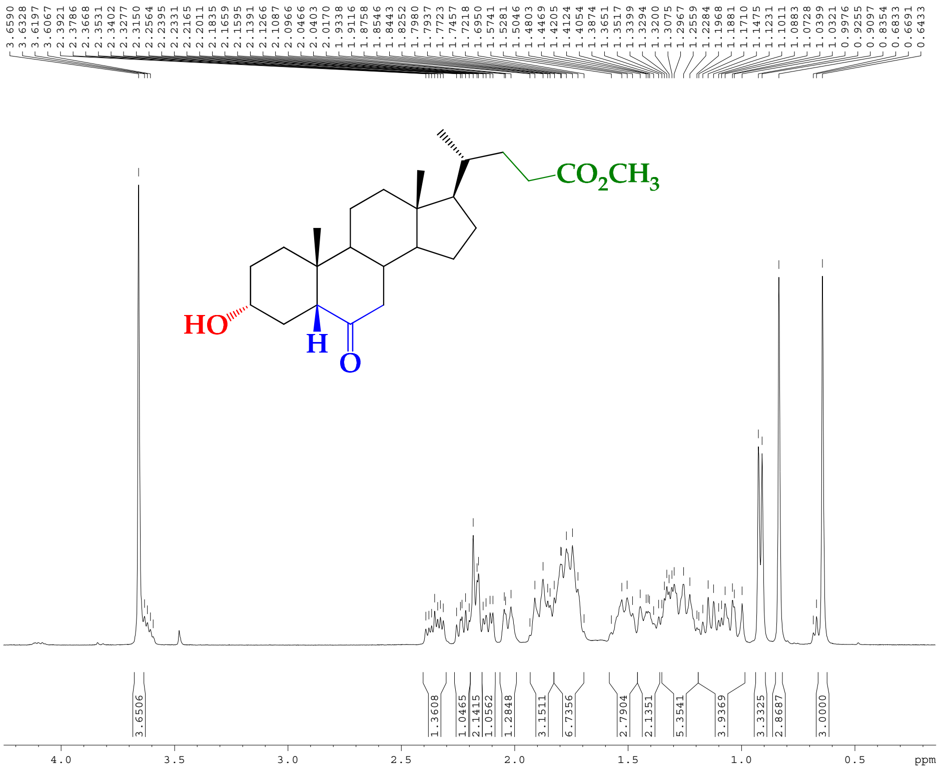

Figure S6. ^{1}H-NMR of Methyl 3α-hydroxy-6-oxo-5β-cholan-24-oate (**7**).

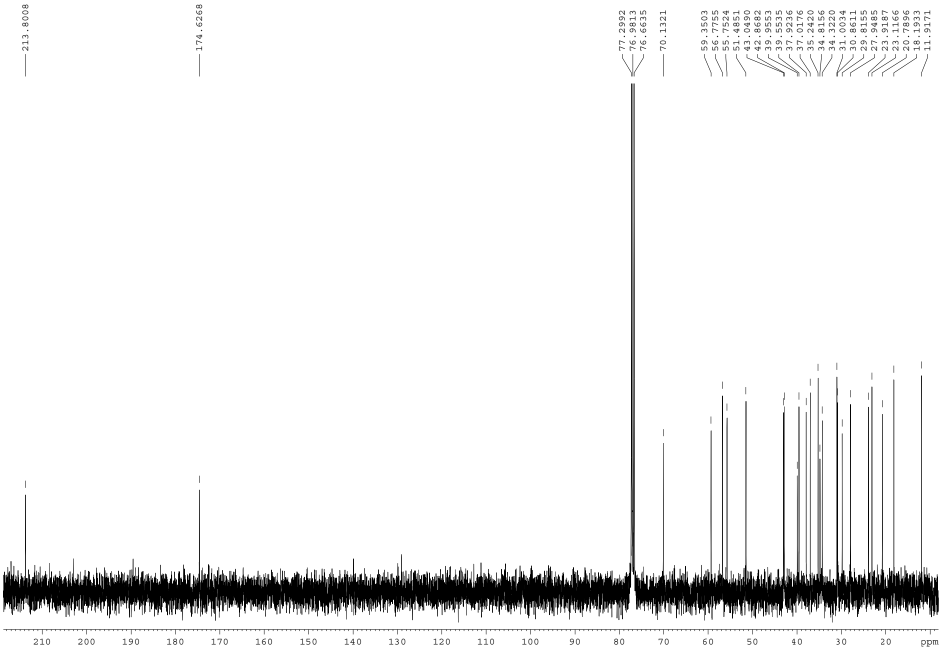

Figure S7. ^{13}C-NMR of Methyl 3α-hydroxy-6-oxo-5β-cholan-24-oate (**7**).

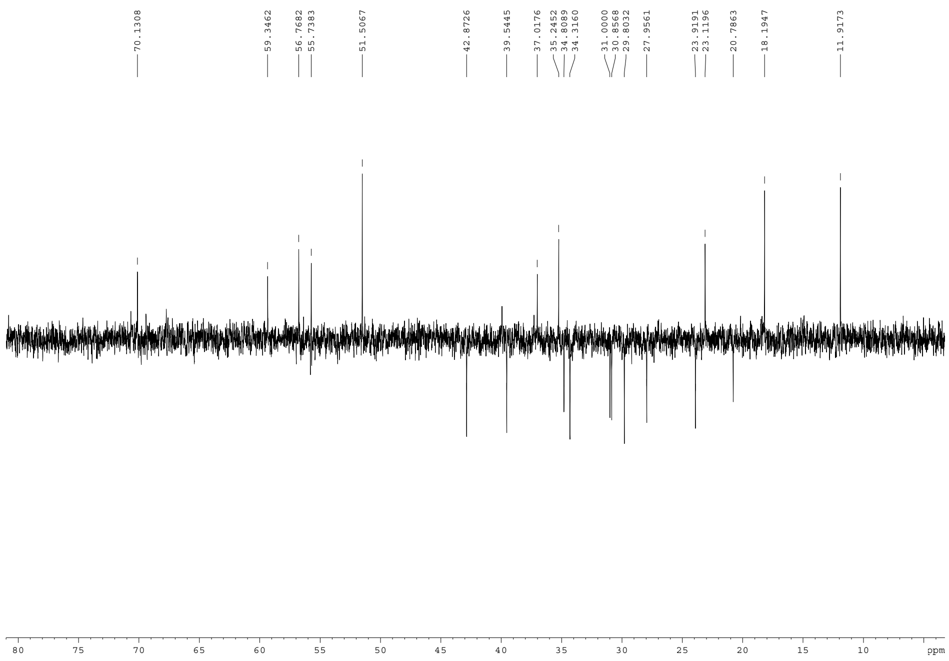

Figure S8. ^{13}C DEPT-135 NMR of Methyl 3α-hydroxy-6-oxo-5β-cholan-24-oate (**7**).

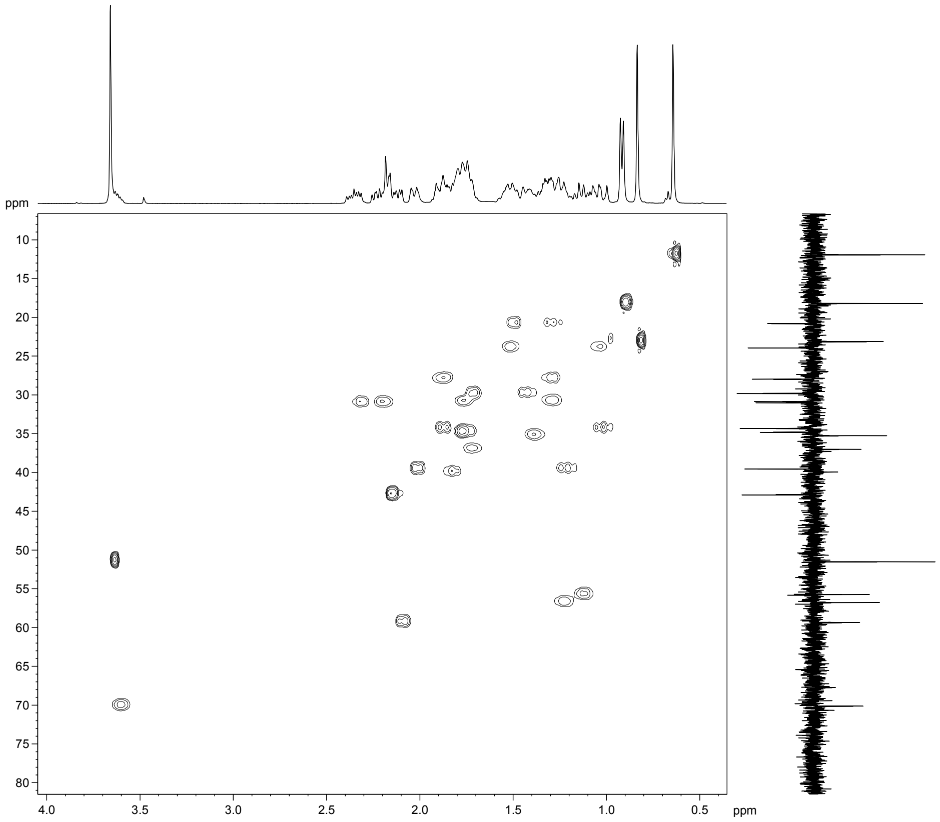

Figure S9. ^{1}H-^{13}C 2D HSQC of Methyl 3α-hydroxy-6-oxo-5β-cholan-24-oate (**7**).

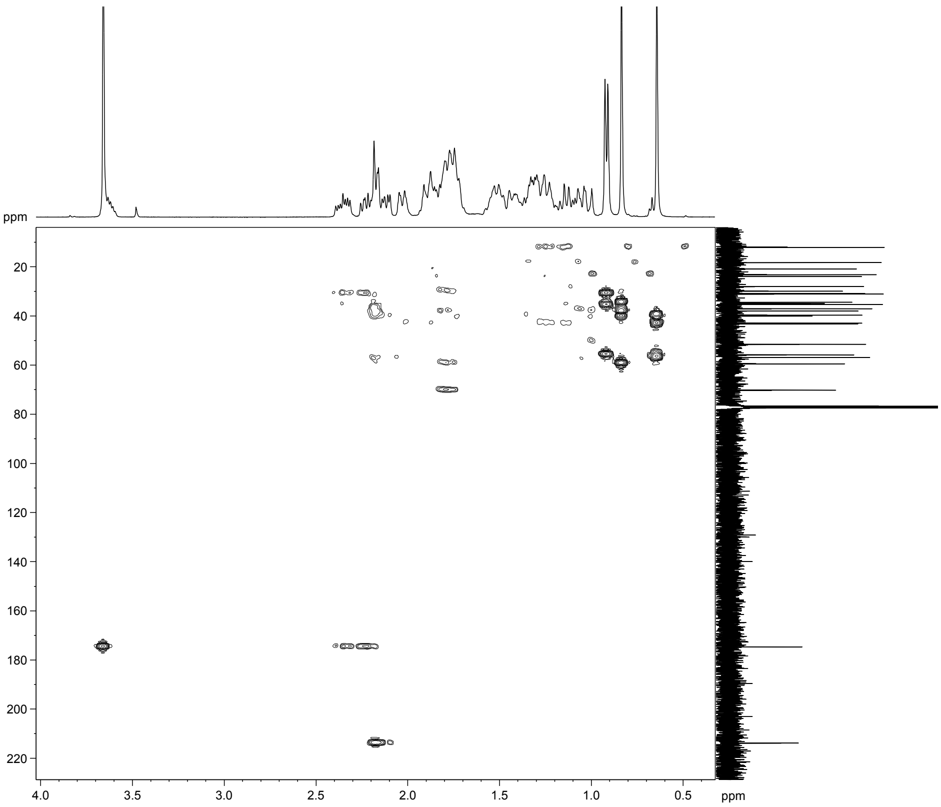

Figure S10. ^{1}H-^{13}C 2D HMBC of Methyl 3α-hydroxy-6-oxo-5β-cholan-24-oate (**7**).

Methyl 3α-hydroxy-6-oxo-5α-cholan-24-oate (**8**)

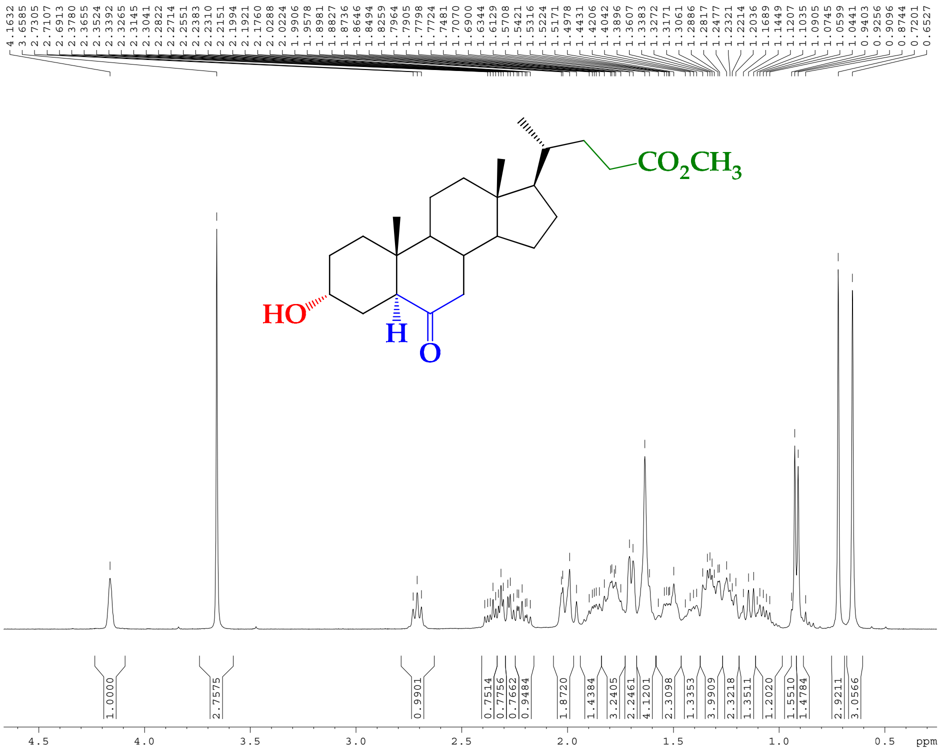

Figure S11. ^{1}H-NMR of Methyl 3α-hydroxy-6-oxo-5α-cholan-24-oate (**8**).

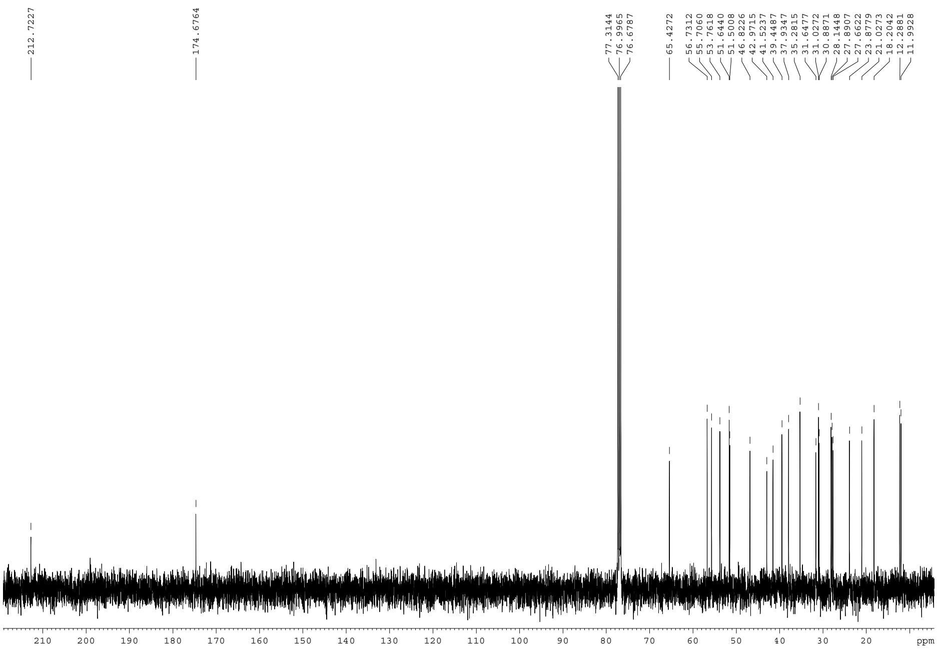

Figure S12. ^{13}C-NMR of Methyl 3α-hydroxy-6-oxo-5α-cholan-24-oate (**8**).

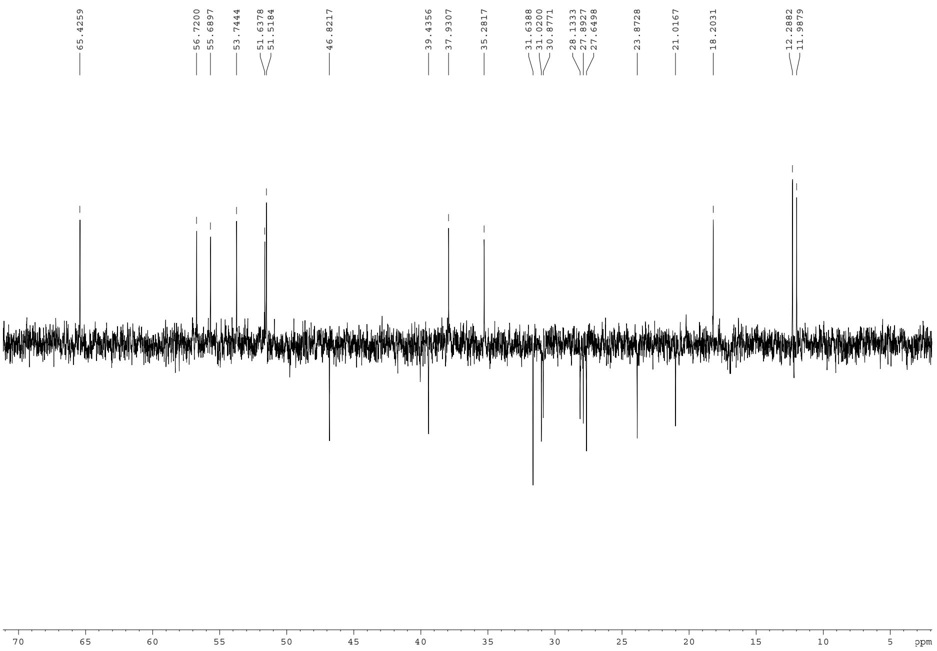

Figure S13. ^{13}C DEPT-135 NMR of Methyl 3α-hydroxy-6-oxo-5α-cholan-24-oate (**8**).

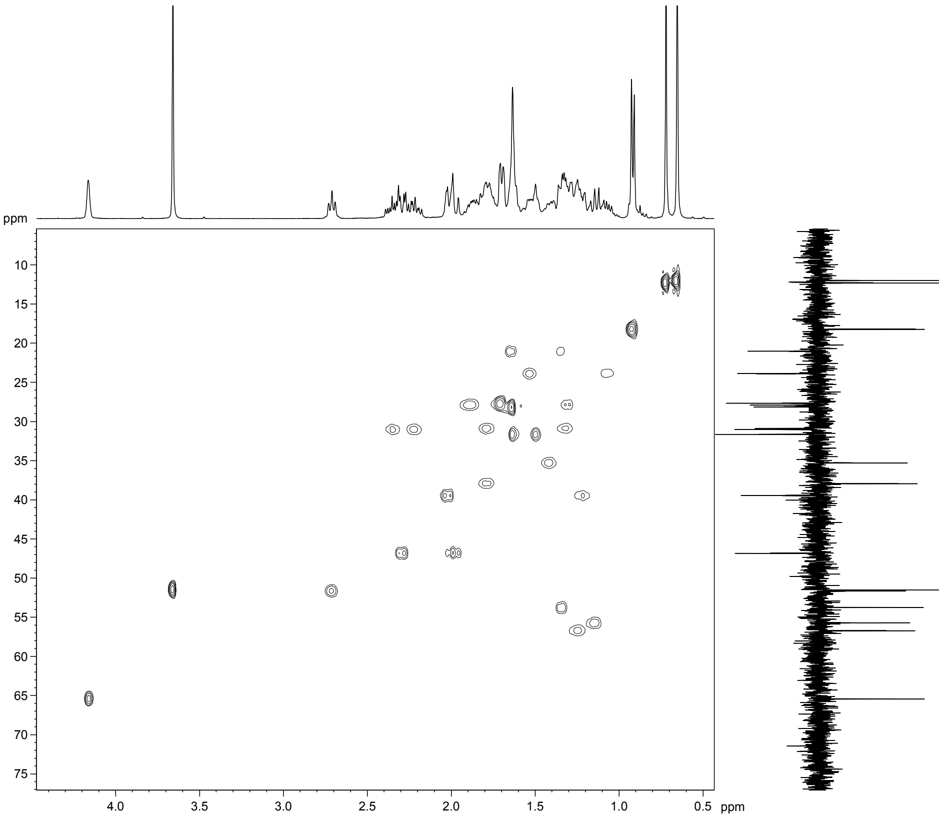

Figure S14. ^{1}H-^{13}C HSQC of Methyl 3α-hydroxy-6-oxo-5α-cholan-24-oate (**8**).

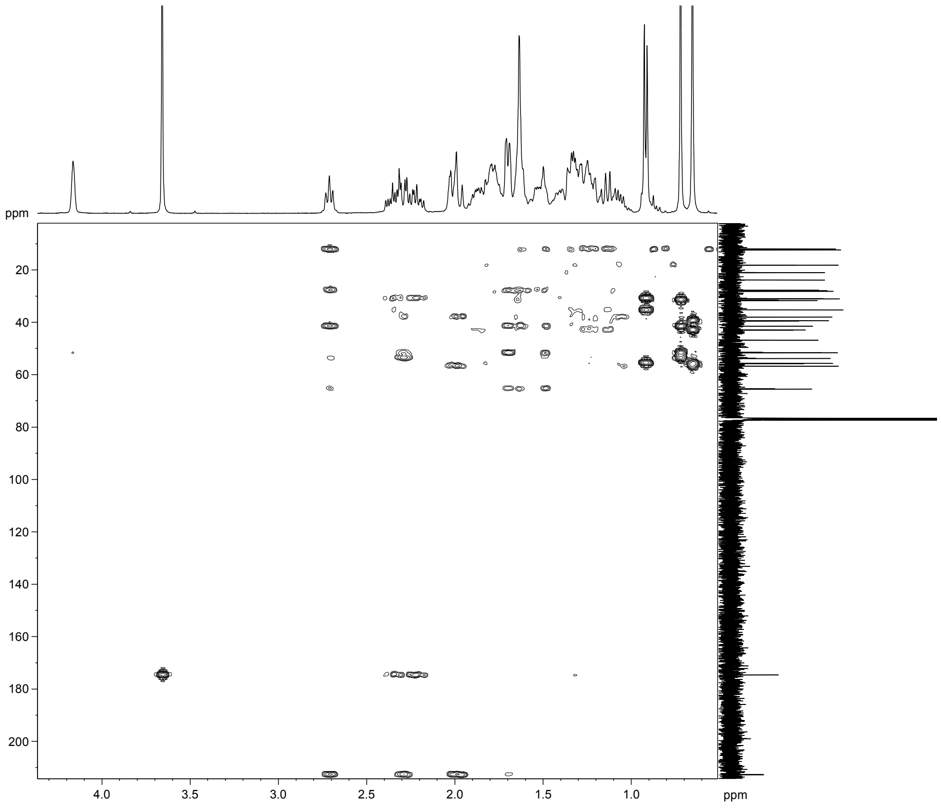

Figure S15. ^{1}H-^{13}C 2D HMBC of Methyl 3α-hydroxy-6-oxo-5α-cholan-24-oate (**8**).

Methyl 3α-methanesulfonyl-6-oxo-5α-cholan-24-oate (**9**)

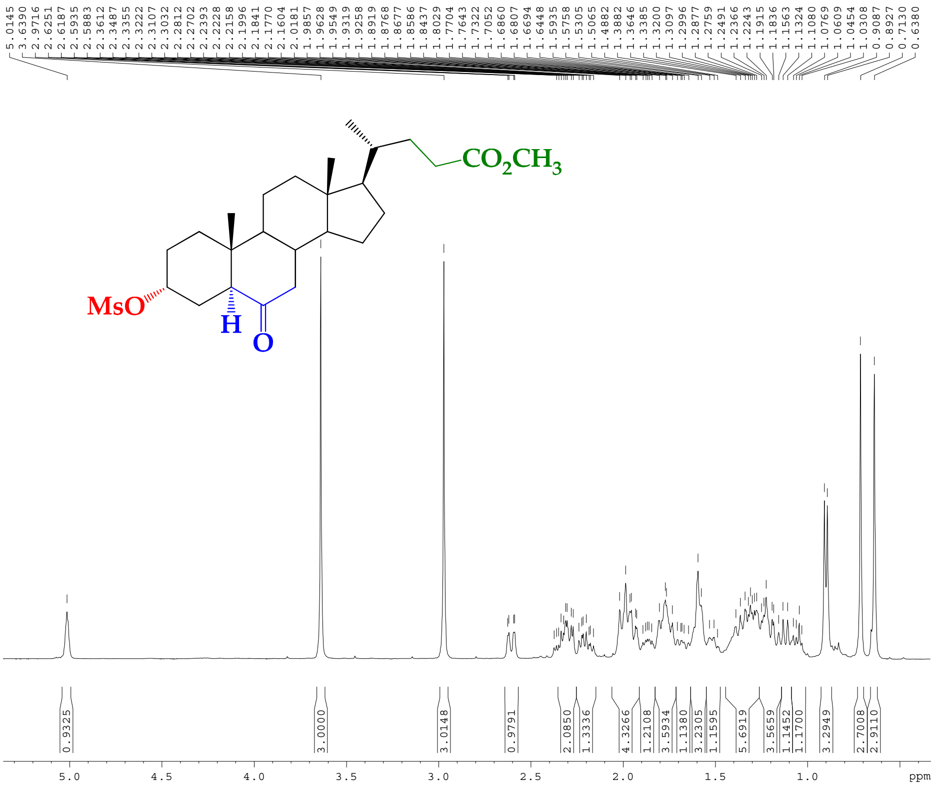

Figure S16. ^{1}H-NMR of Methyl 3α-methanesulfonyl-6-oxo-5α-cholan-24-oate (**9**).

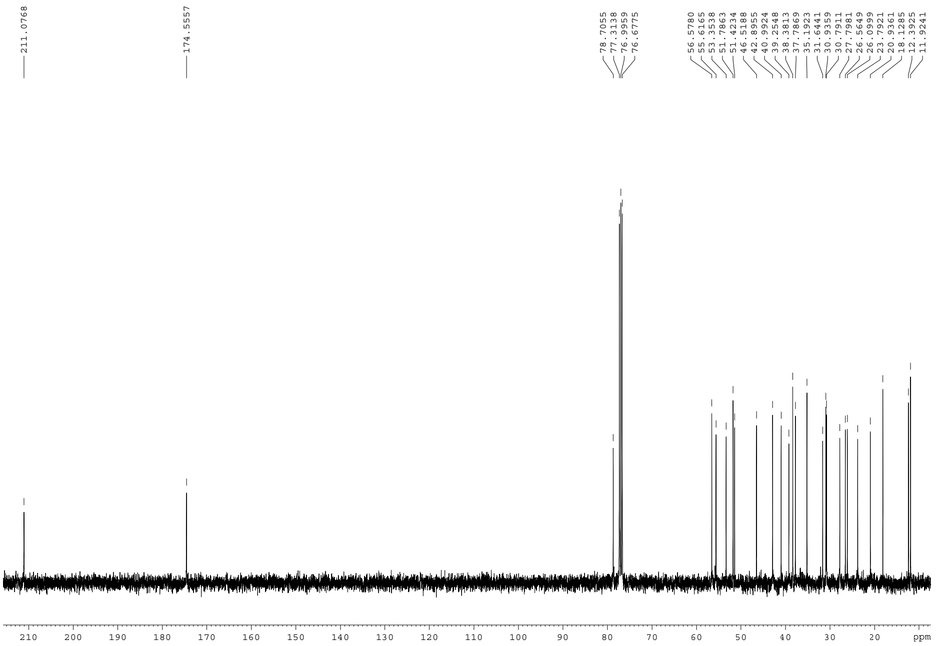

Figure S17. ^{13}C-NMR of Methyl 3α-methanesulfonyl-6-oxo-5α-cholan-24-oate (**9**).

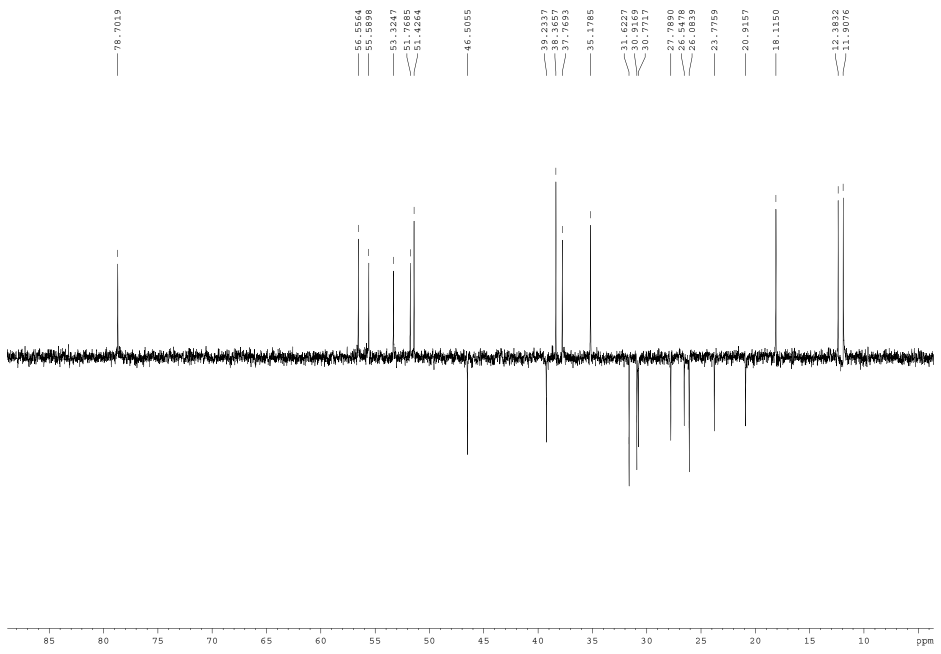

Figure S18. ^{13}C DEPT-135 NMR of Methyl 3α-methanesulfonyl-6-oxo-5α-cholan-24-oate (**9**).

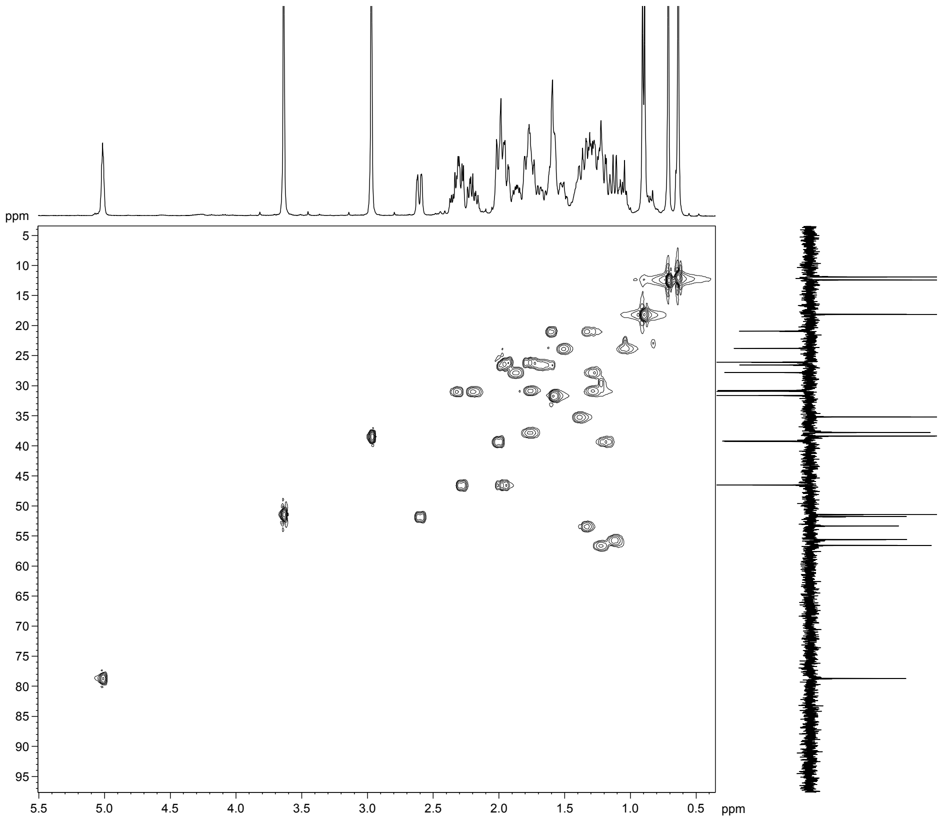

Figure S19. ^{1}H-^{13}C HSQC of Methyl 3α-methanesulfonyl-6-oxo-5α-cholan-24-oate (**9**).

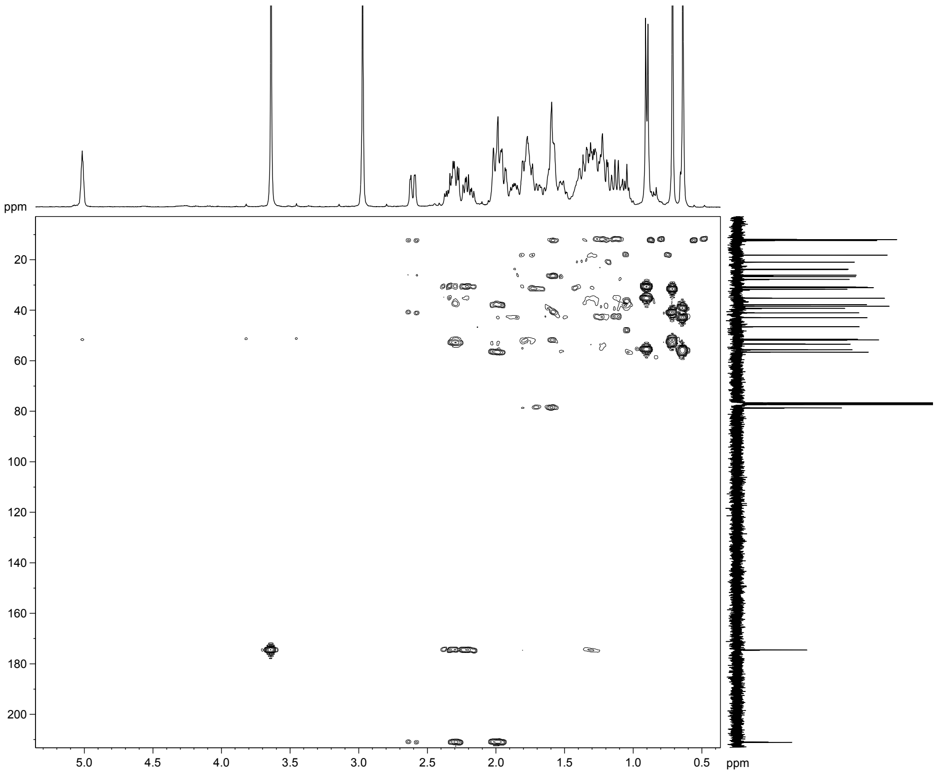

Figure S20. ^{1}H-^{13}C 2D HMBC of Methyl 3α-methanesulfonyl-6-oxo-5α-cholan-24-oate (**9**).

Methyl 2-en-6-oxo-5α-cholan-24-oate (**10**)

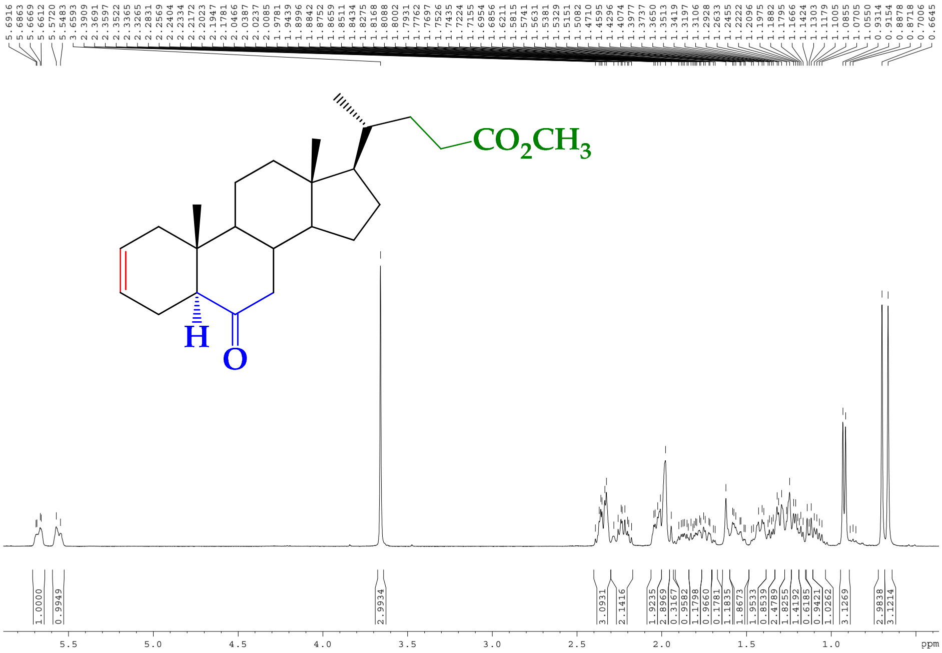

Figure S21. ^{1}H-NMR of Methyl 2-en-6-oxo-5α-cholan-24-oate (**10**).

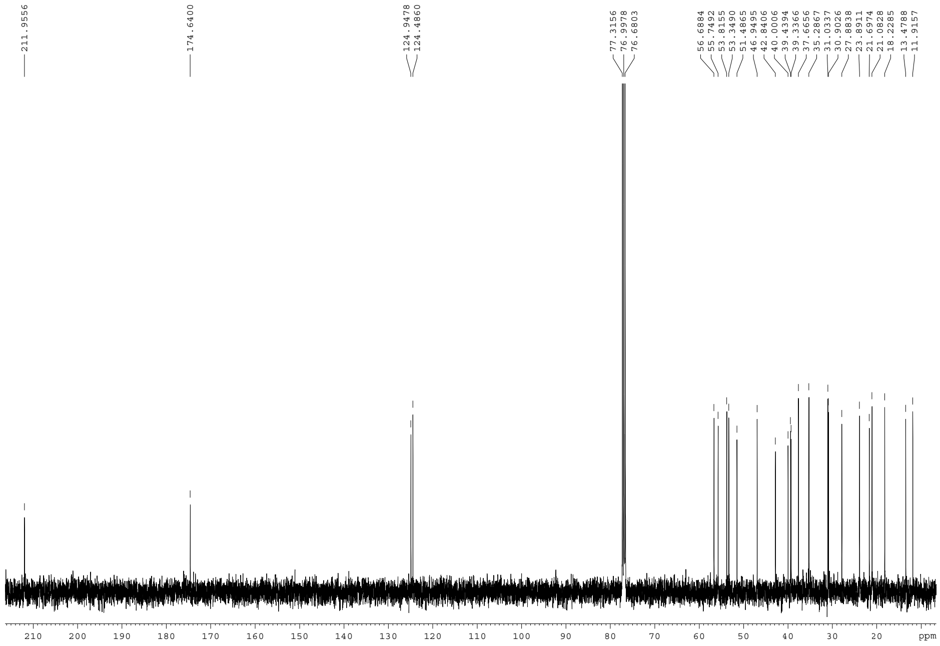

Figure S22. ^{13}C-NMR of Methyl 2-en-6-oxo-5α-cholan-24-oate (**10**).

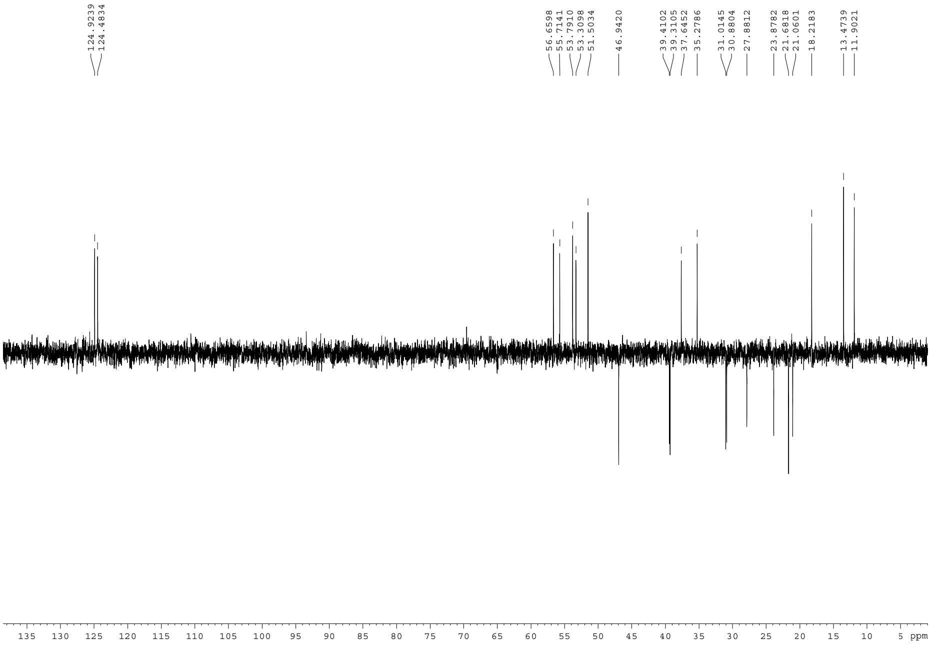

Figure S23. ^{13}C DEPT-135 NMR of Methyl 2-en-6-oxo-5α-cholan-24-oate (**10**).

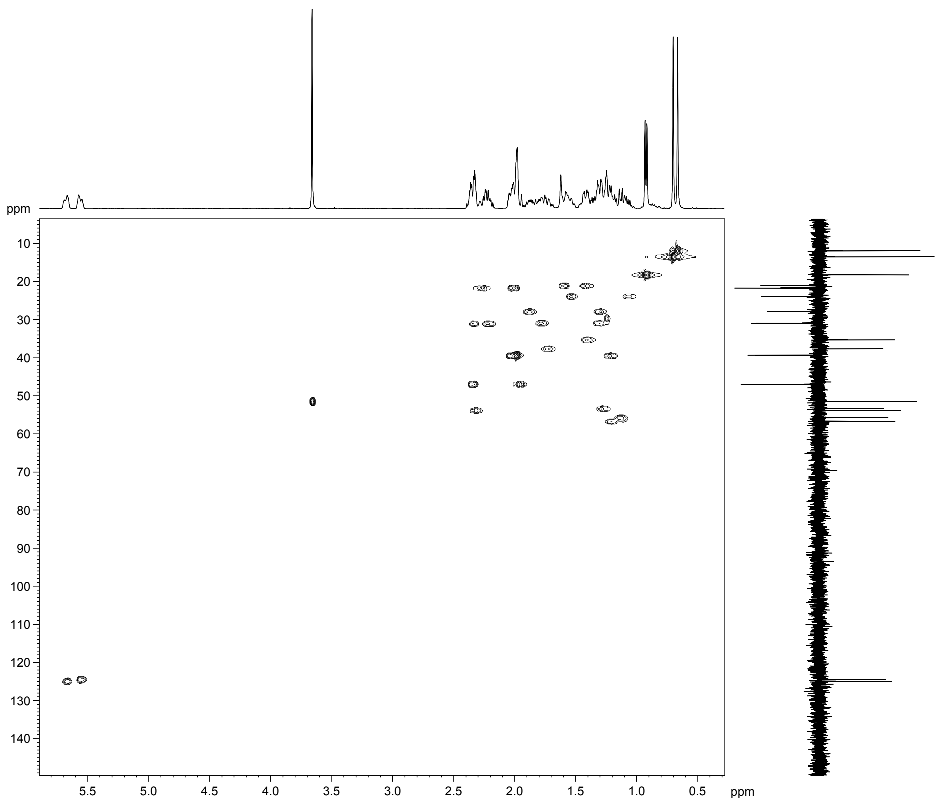

Figure S24. ^{1}H-^{13}C HSQC of Methyl 2-en-6-oxo-5α-cholan-24-oate (**10**).

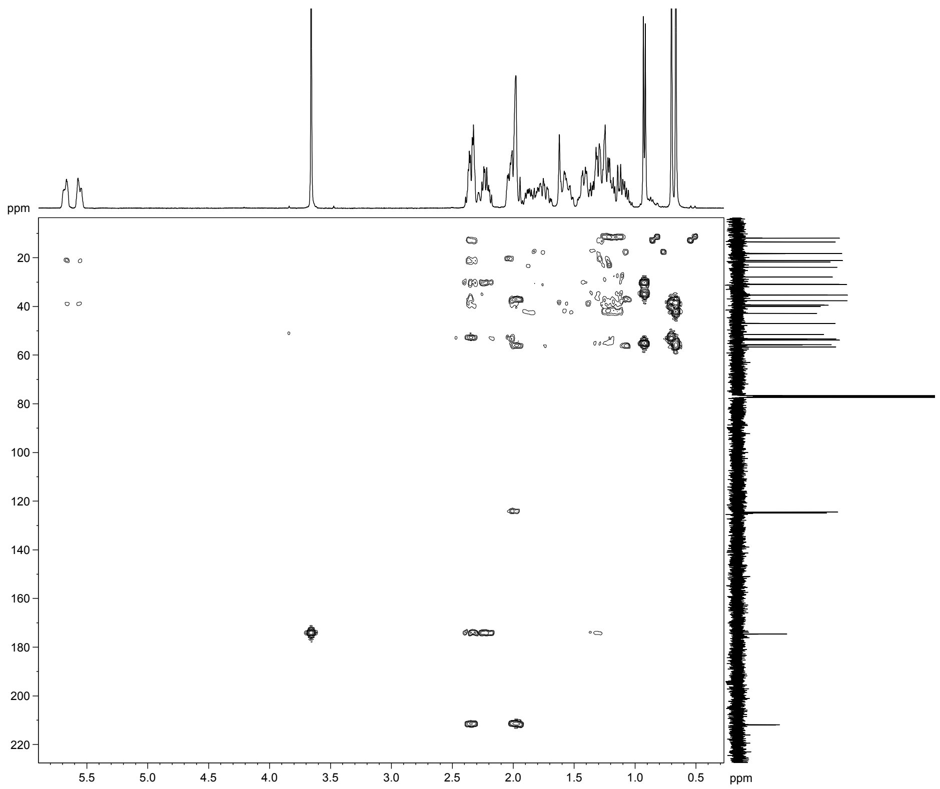

Figure S25. ^{1}H-^{13}C 2D HMBC of Methyl 2-en-6-oxo-5α-cholan-24-oate (**10**).

Methyl 2α,3α-dihydroxy-6-oxo-5α-cholan-24-oate (**11**)

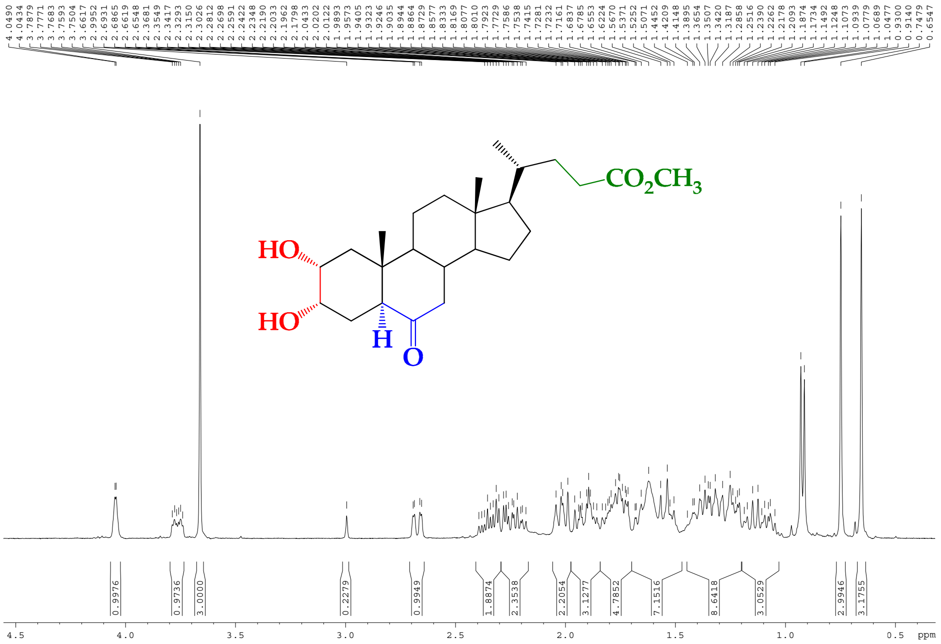

Figure S26. ^{1}H-NMR of Methyl 2α,3α-dihydroxy-6-oxo-5α-cholan-24-oate (**11**).

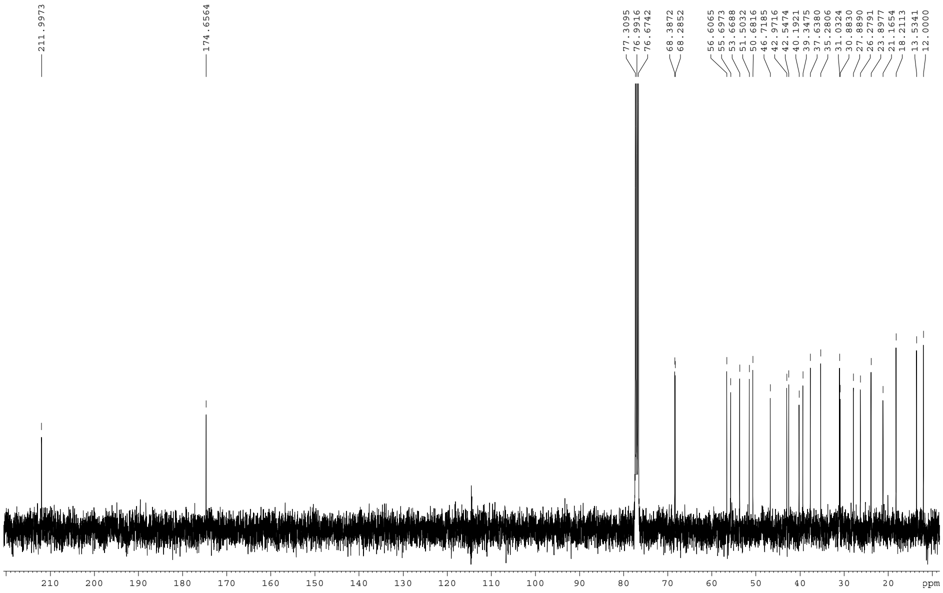

Figure S27. ^{13}C-NMR of Methyl 2α,3α-dihydroxy-6-oxo-5α-cholan-24-oate (**11**).

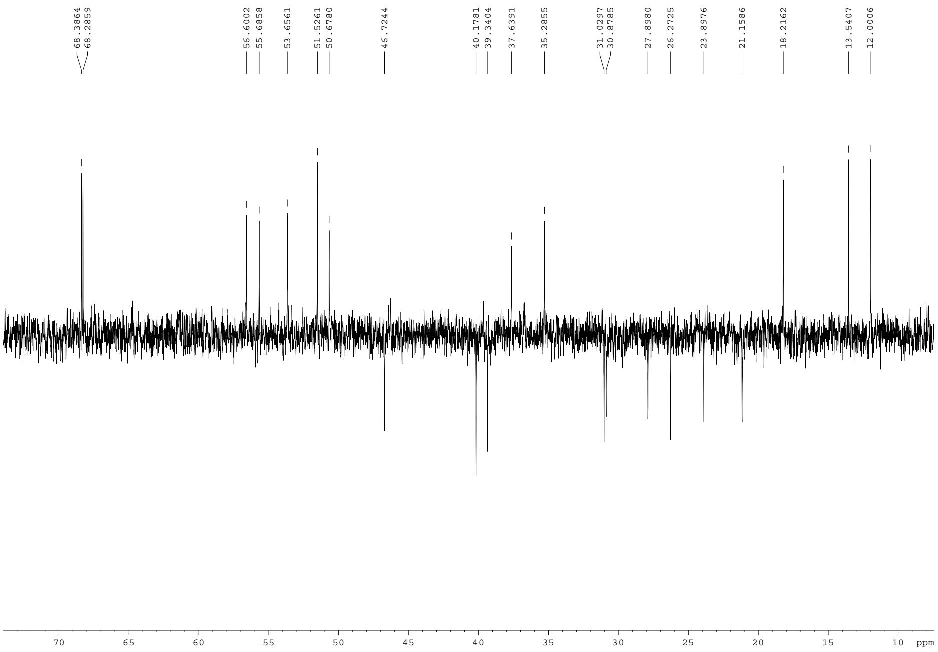

Figure S28. ^{13}C DEPT-135 NMR of Methyl 2α,3α-dihydroxy-6-oxo-5α-cholan-24-oate (**11**).

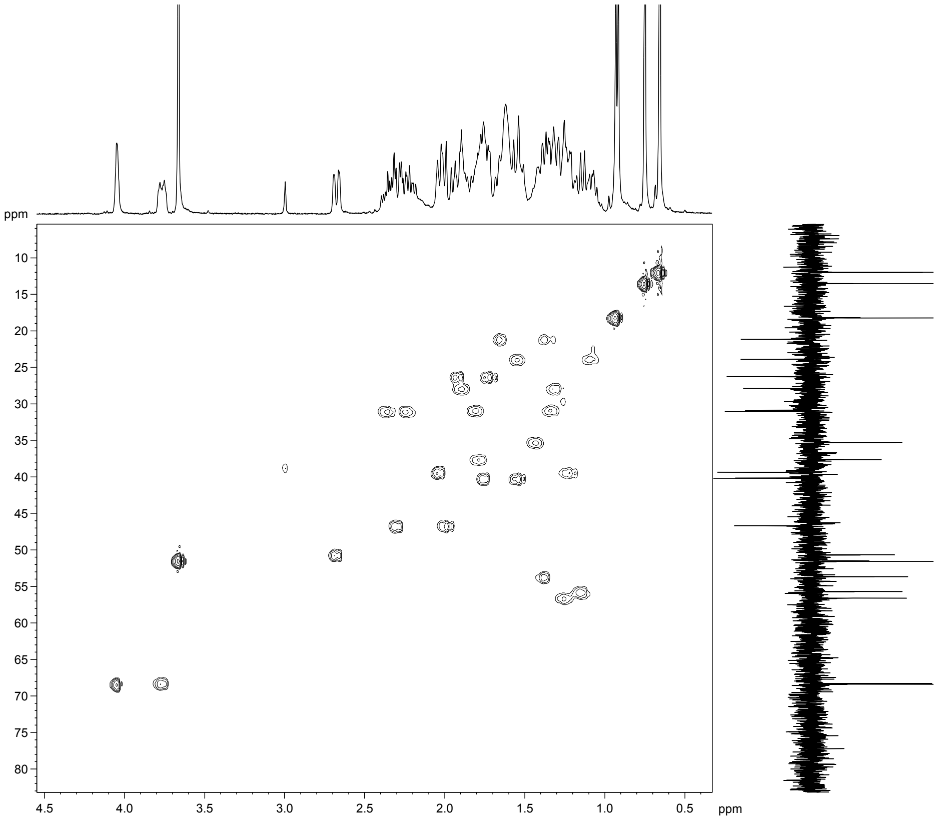

Figure S29. ^{1}H-^{13}C HSQC of Methyl 2α,3α-dihydroxy-6-oxo-5α-cholan-24-oate (**11**).

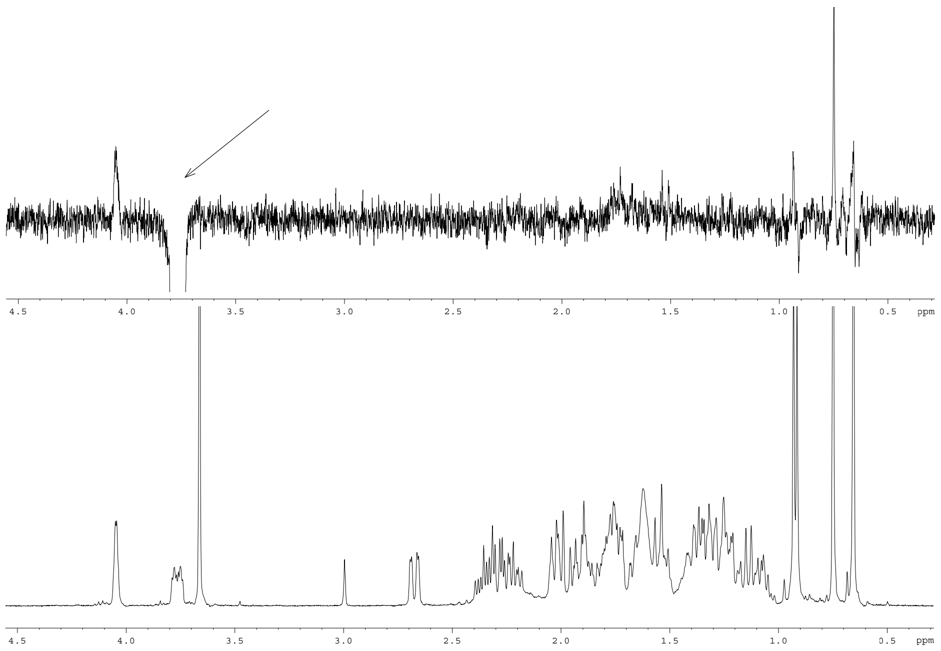

Figure S30. ^{1}H 1D Selective NOESY NMR of Methyl 2α,3α-dihydroxy-6-oxo-5α-cholan-24-oate (**11**).

Methyl 3α-acetoxy-6-oxo-5α-cholan-24-oate (**12**)

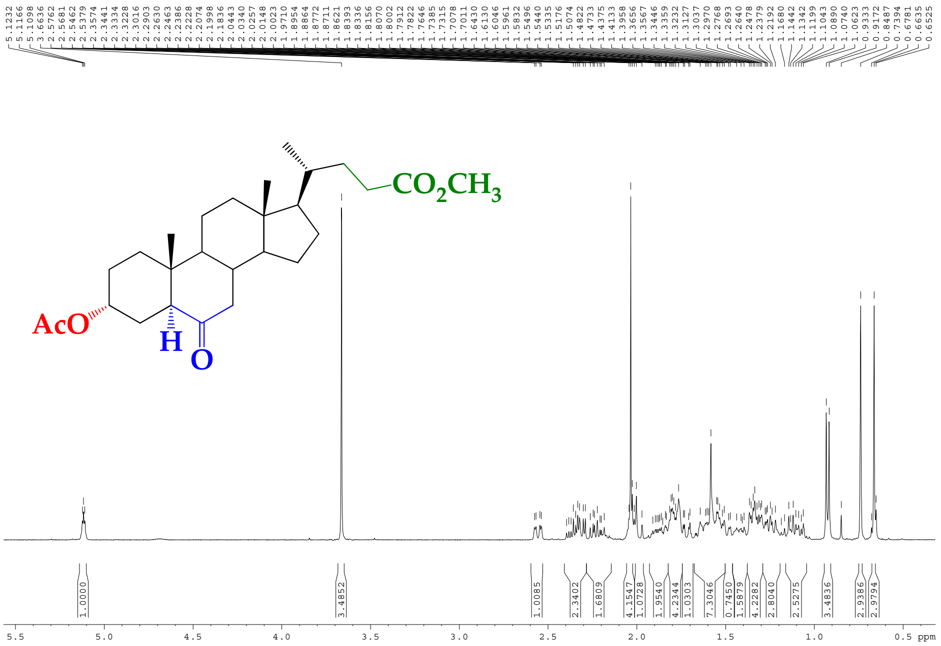

Figure S31. ^{1}H-NMR of Methyl 3α-acetoxy-6-oxo-5α-cholan-24-oate (**12**).

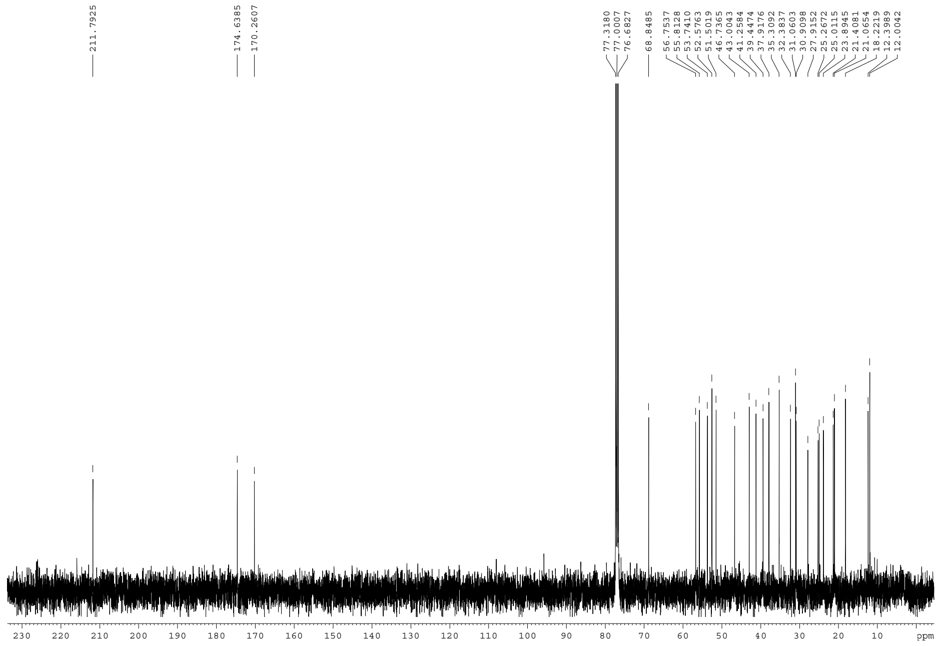

Figure S32. ^{13}C-NMR of Methyl 3α-acetoxy-6-oxo-5α-cholan-24-oate (**12**).

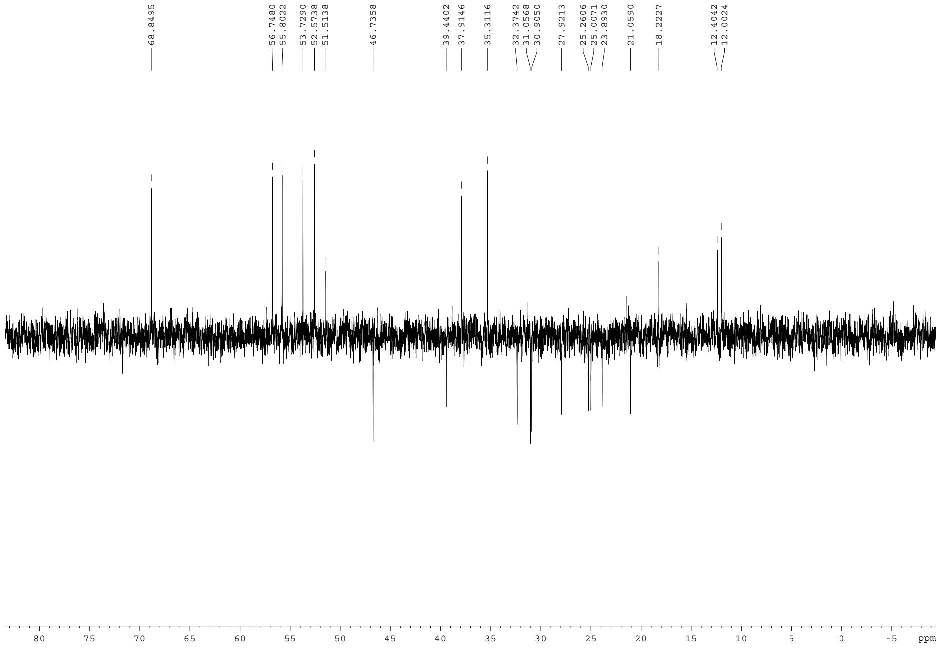

Figure S33. ^{13}C DEPT-135 NMR of Methyl 3α-acetoxy-6-oxo-5α-cholan-24-oate (**12**).

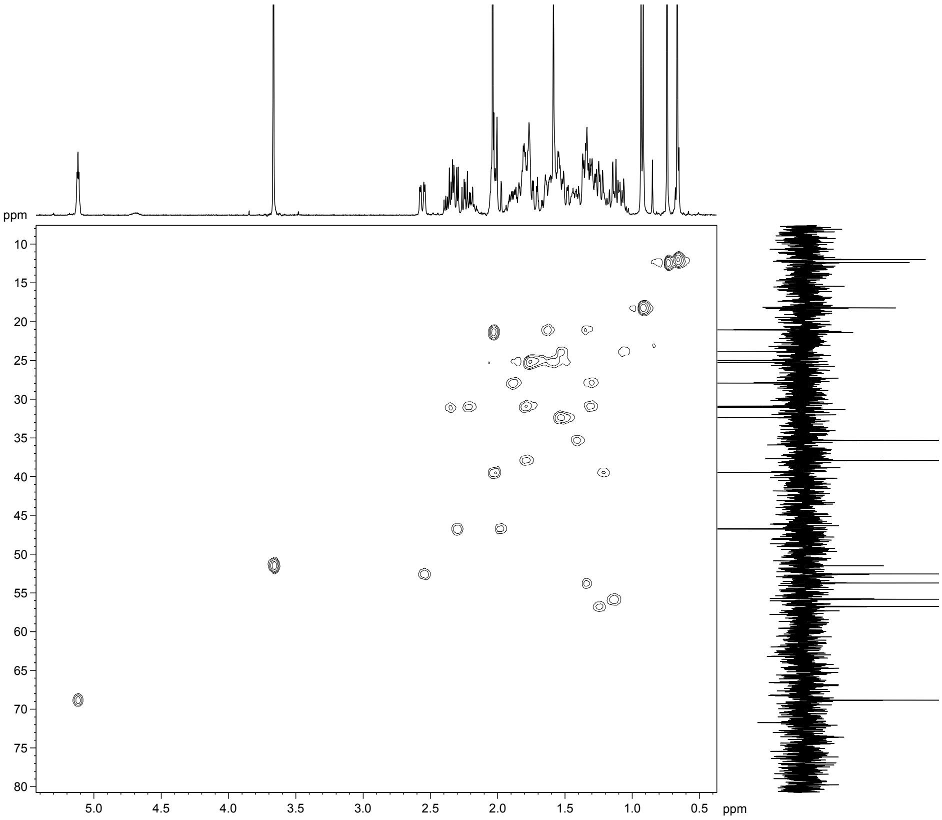

Figure S34. ^{1}H-^{13}C HSQC of Methyl 3α-acetoxy-6-oxo-5α-cholan-24-oate (**12**).

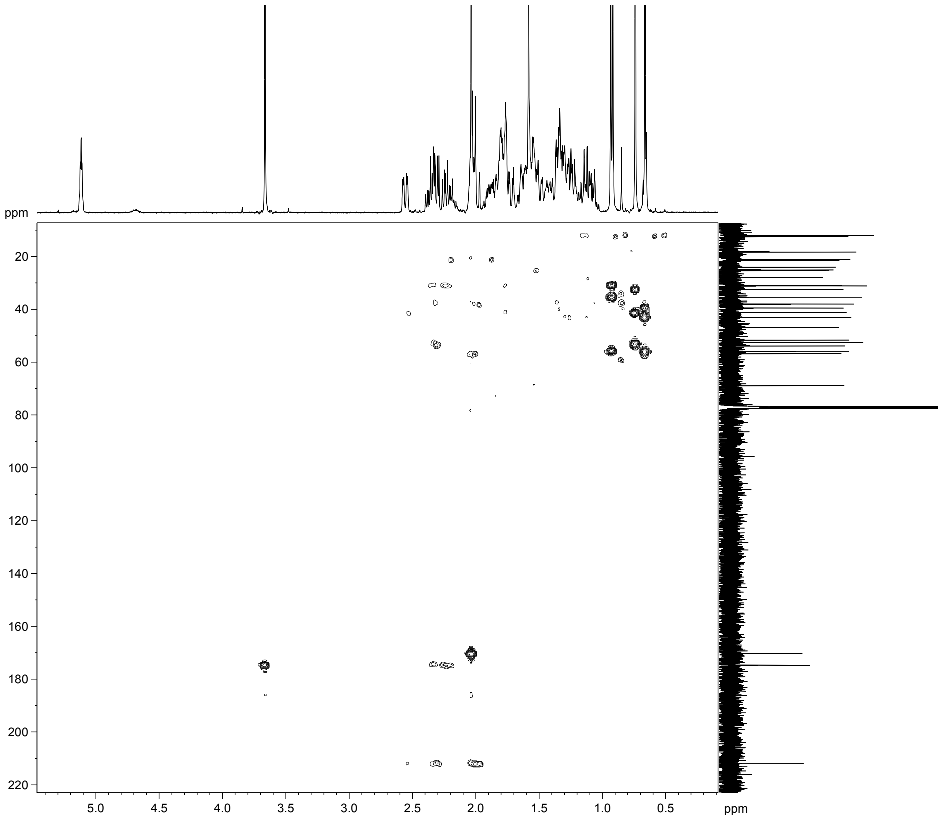

Figure S35. ^{1}H-^{13}C 2D HMBC of Methyl 3α-acetoxy-6-oxo-5α-cholan-24-oate (**12**).

Acid 3α-hydroxy-6-oxo-5α-cholan-24-oic (**13**)

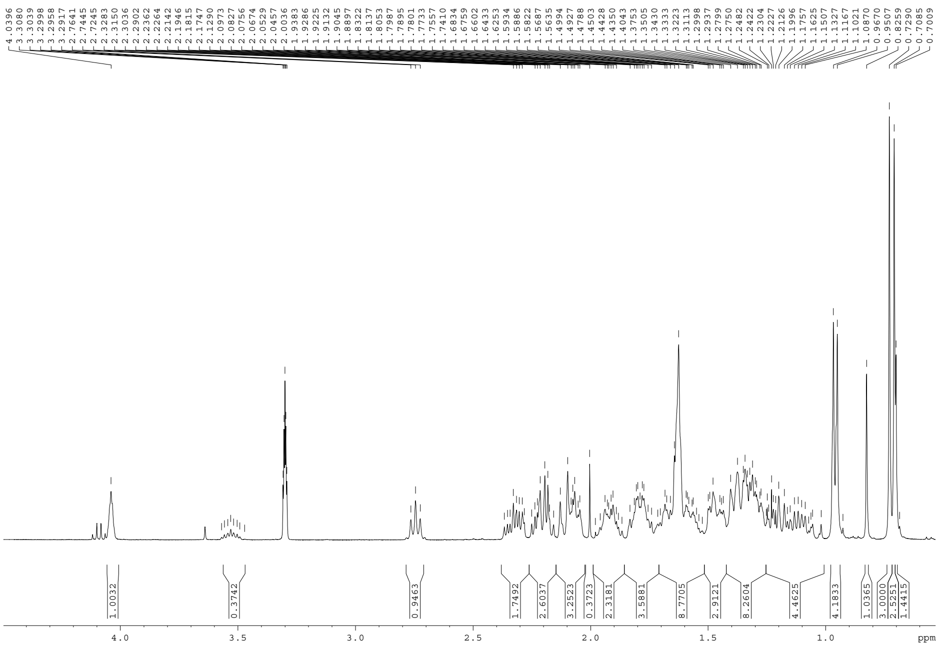

Figure S36. ^{1}H-NMR of Acid 3α-hydroxy-6-oxo-5α-cholan-24-oic (**13**).

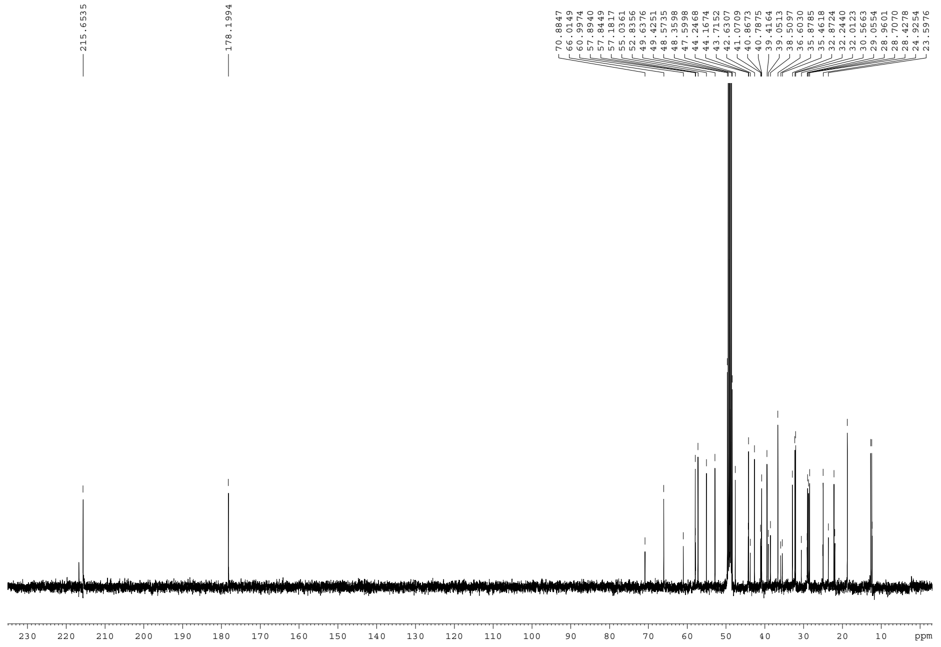

Figure S37. ^{13}C-NMR of Acid 3α-hydroxy-6-oxo-5α-cholan-24-oic (**13**).

Acid 3α-acetoxy-6-oxo-5α-cholan-24-oic (**14**)

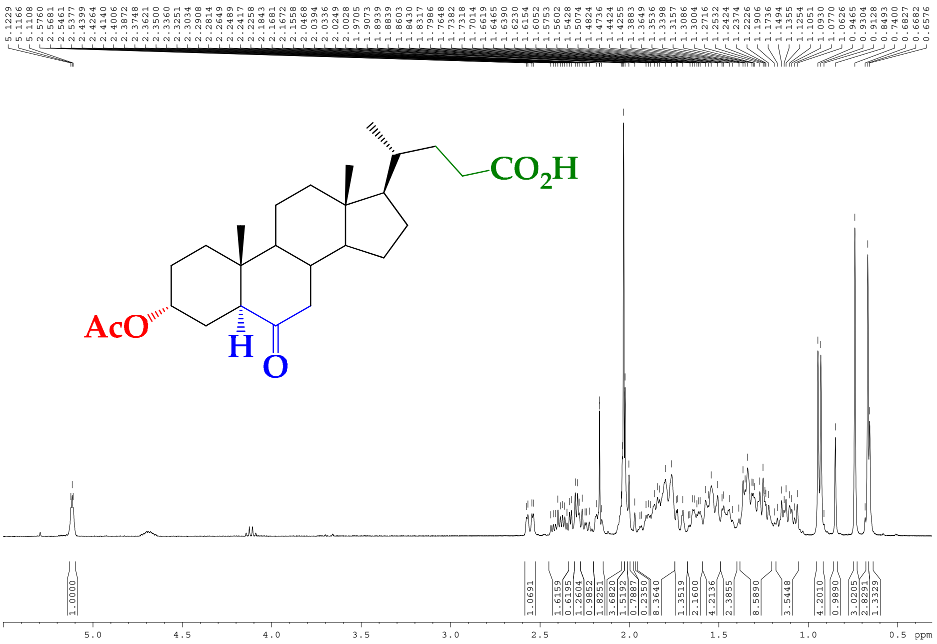

Figure S38. ^{1}H-NMR of Acid 3*α*-acetoxy-6-oxo-5*α*-cholan-24-oic (**14**).

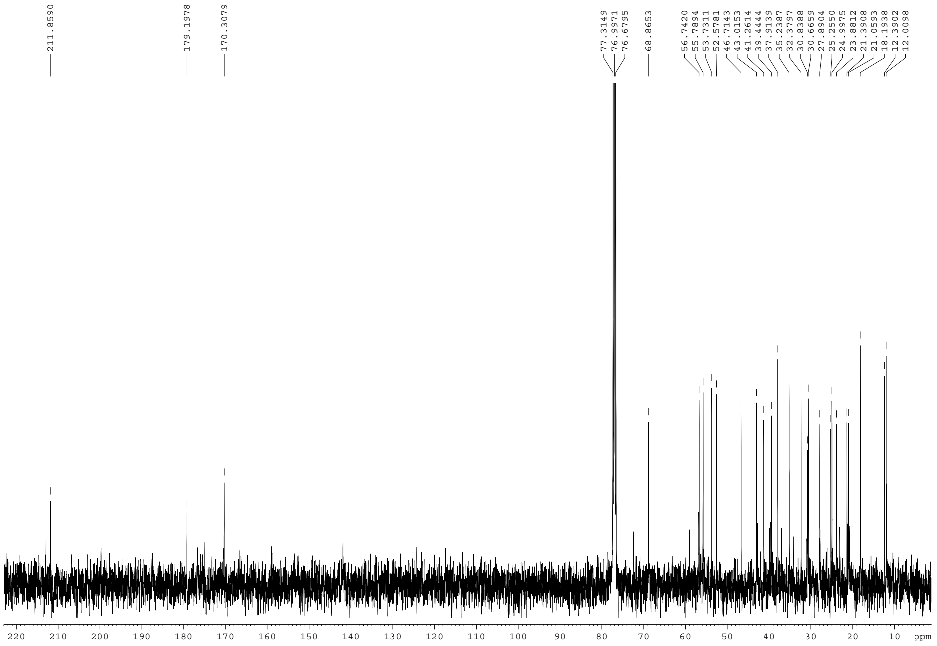

Figure S39. ^{13}C-NMR of Acid 3*α*-acetoxy-6-oxo-5*α*-cholan-24-oic (**14**).

Methyl 3α-hydroxy-6-oxo-7-oxa-5α-cholan-24-oate (**15**)

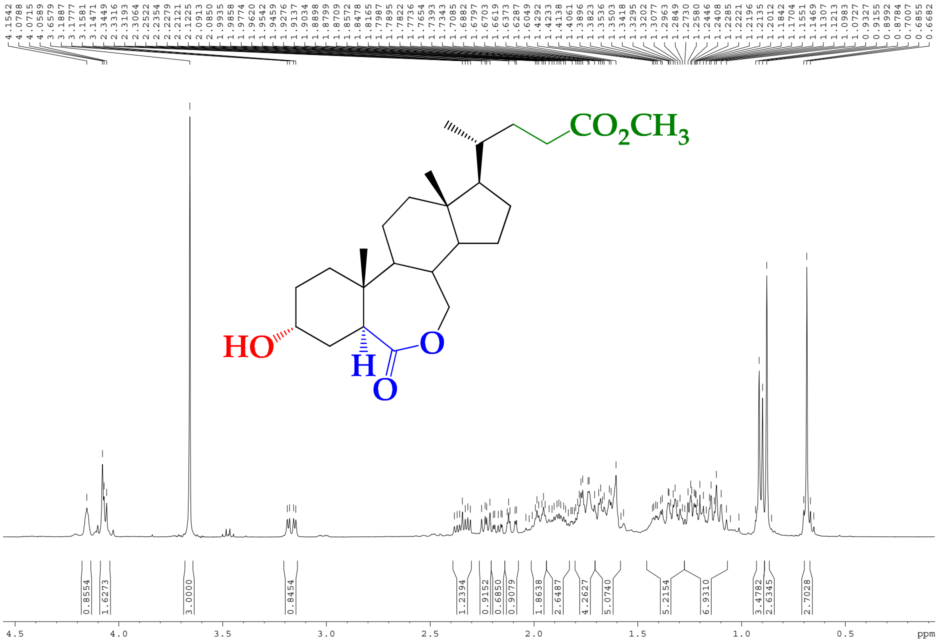

Figure S40. ^{1}H-NMR of Methyl 3α-hydroxy-6-oxo-7-oxa-5α-cholan-24-oate (**15**).

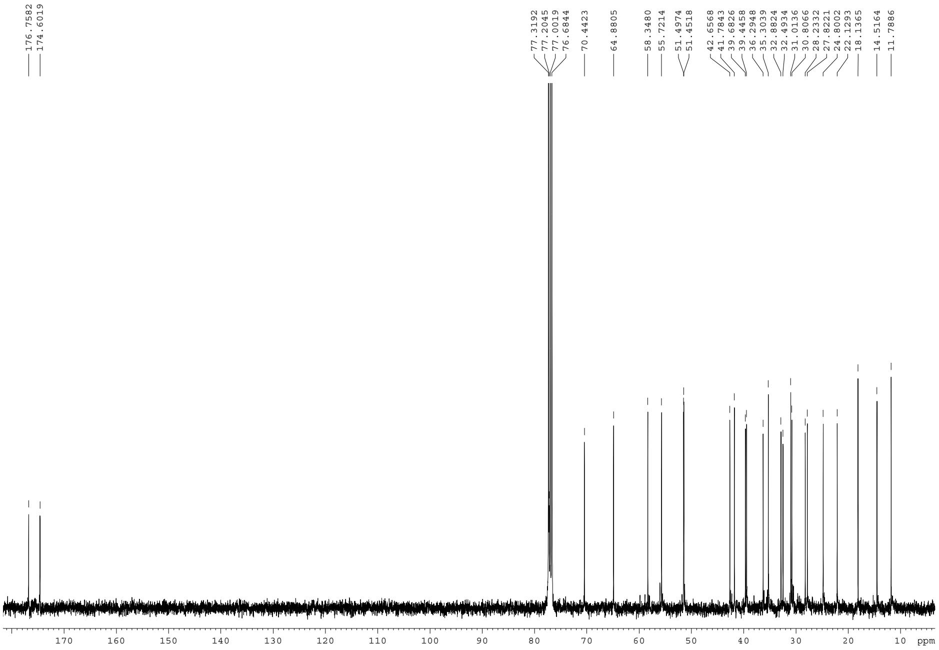

Figure S41. ^{13}C-NMR of Methyl 3α-hydroxy-6-oxo-7-oxa-5α-cholan-24-oate (**15**).

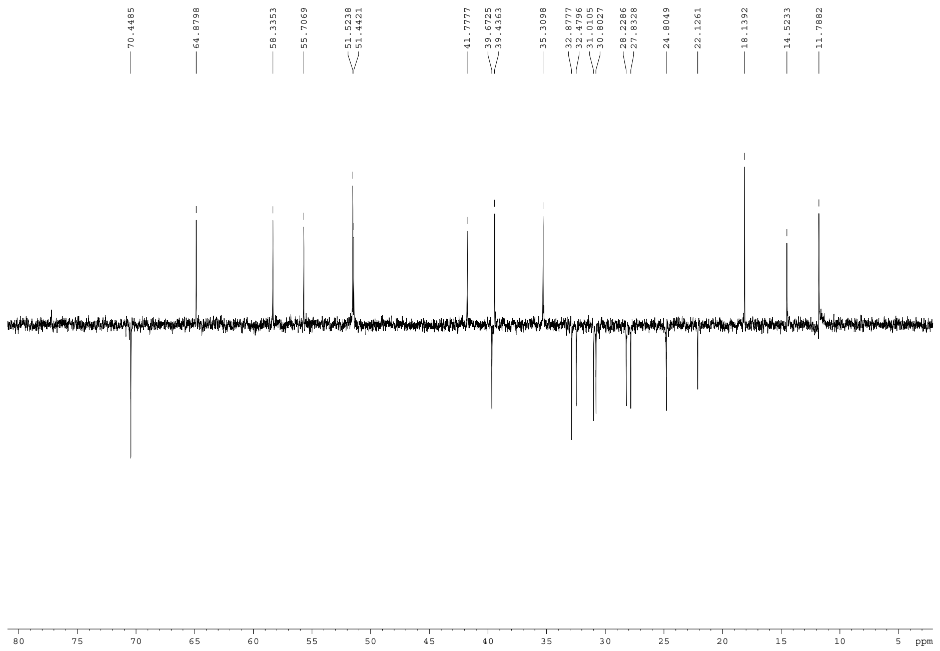

Figure S42. ^{13}C DEPT-135 NMR of Methyl 3α-hydroxy-6-oxo-7-oxa-5α-cholan-24-oate (**15**).

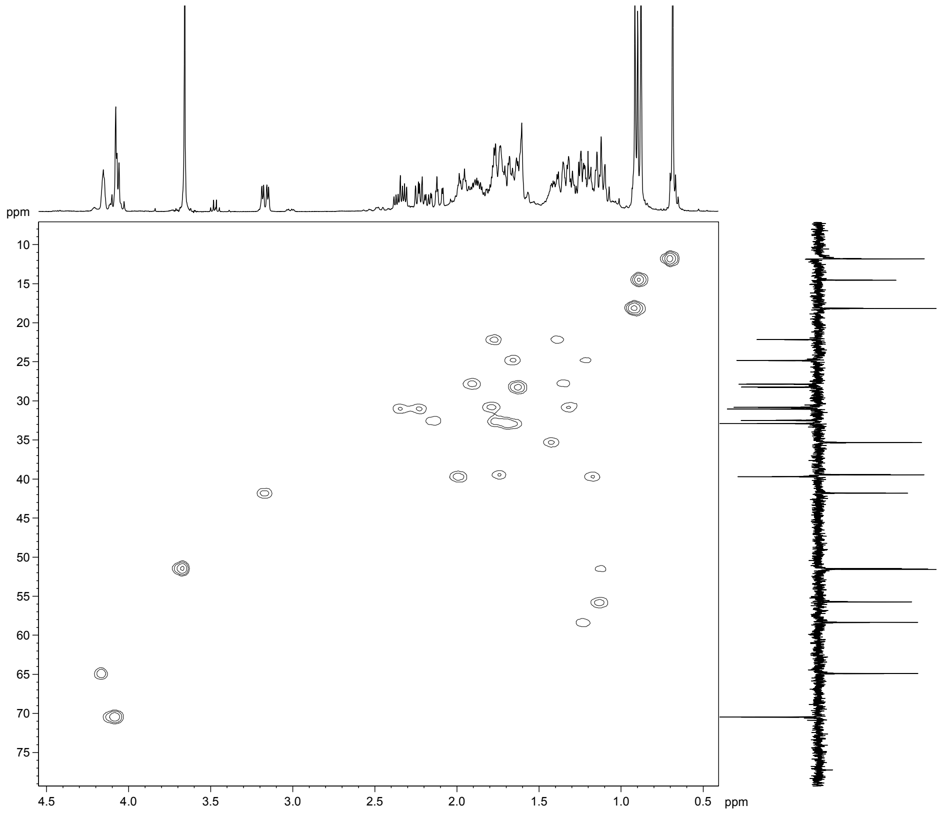

Figure S43. ^{1}H-^{13}C HSQC of Methyl 3α-hydroxy-6-oxo-7-oxa-5α-cholan-24-oate (**15**).

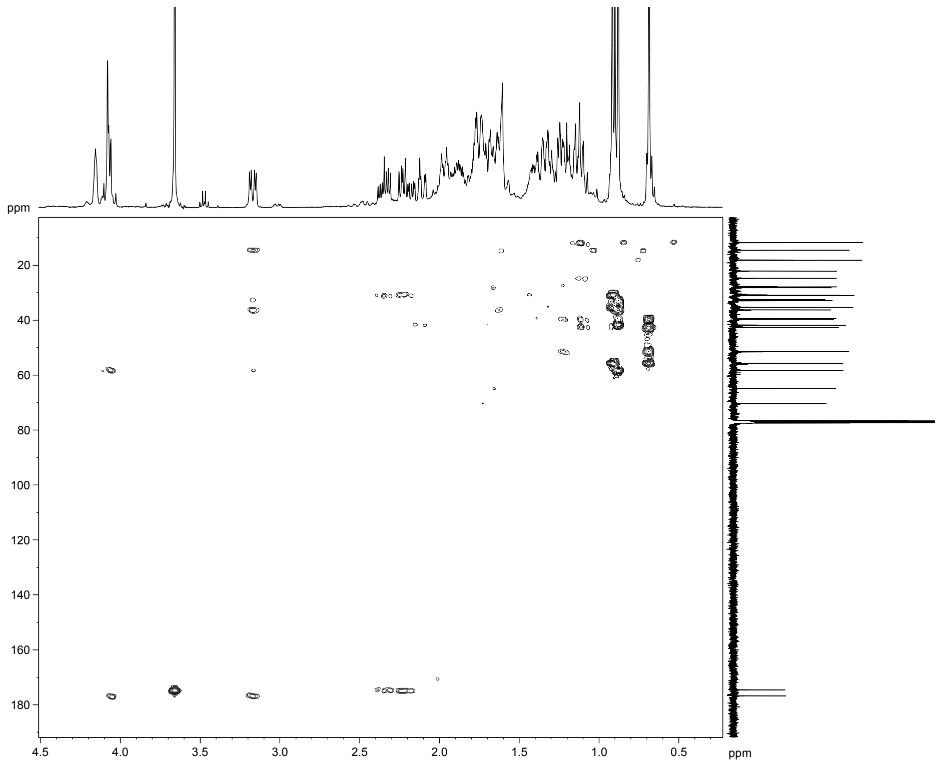

Figure S44. ^{1}H-^{13}C 2D HMBC of Methyl 3α-hydroxy-6-oxo-7-oxa-5α-cholan-24-oate (**15**).

Methyl 3α-hydroxy-6-oxa-7-oxo-5α-cholan-24-oate (**16**)

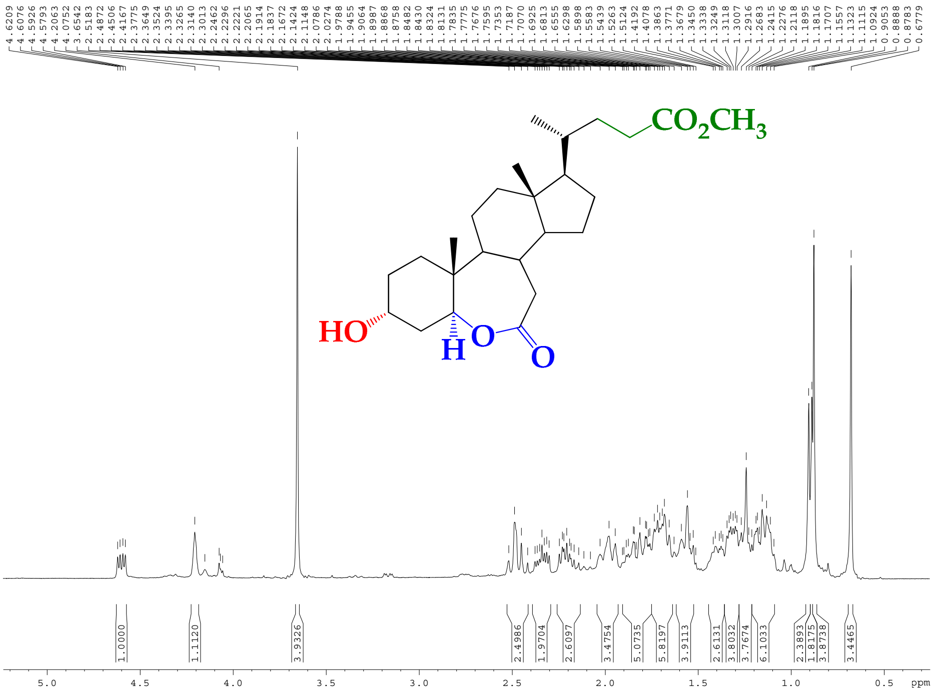

Figure S45. ^{1}H-NMR of Methyl 3α-hydroxy-6-oxa-7-oxo-5α-cholan-24-oate (**16**).

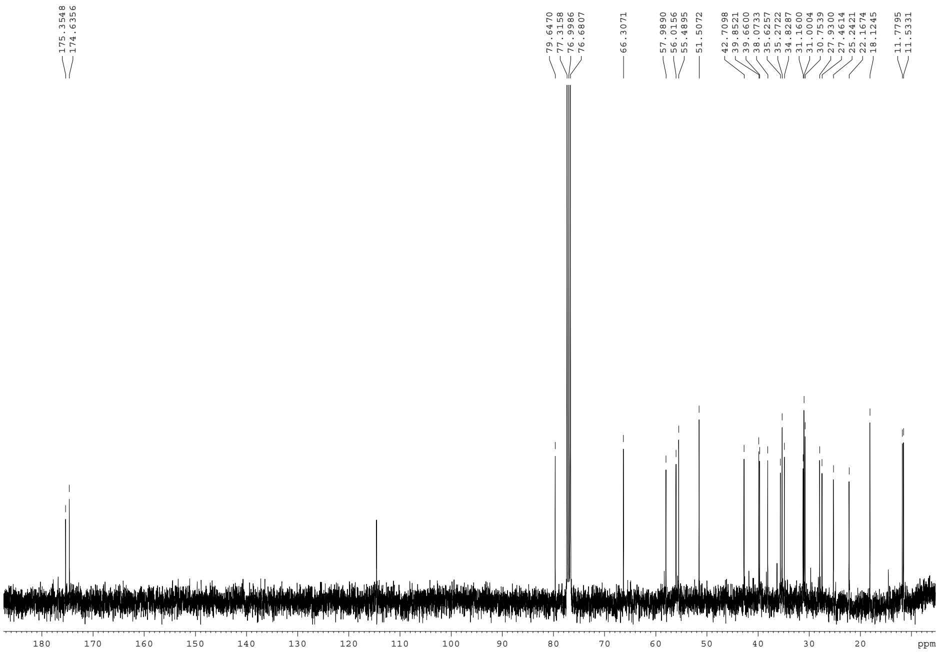

Figure S46. ^{13}C-NMR of Methyl 3α-hydroxy-6-oxa-7-oxo-5α-cholan-24-oate (**16**).

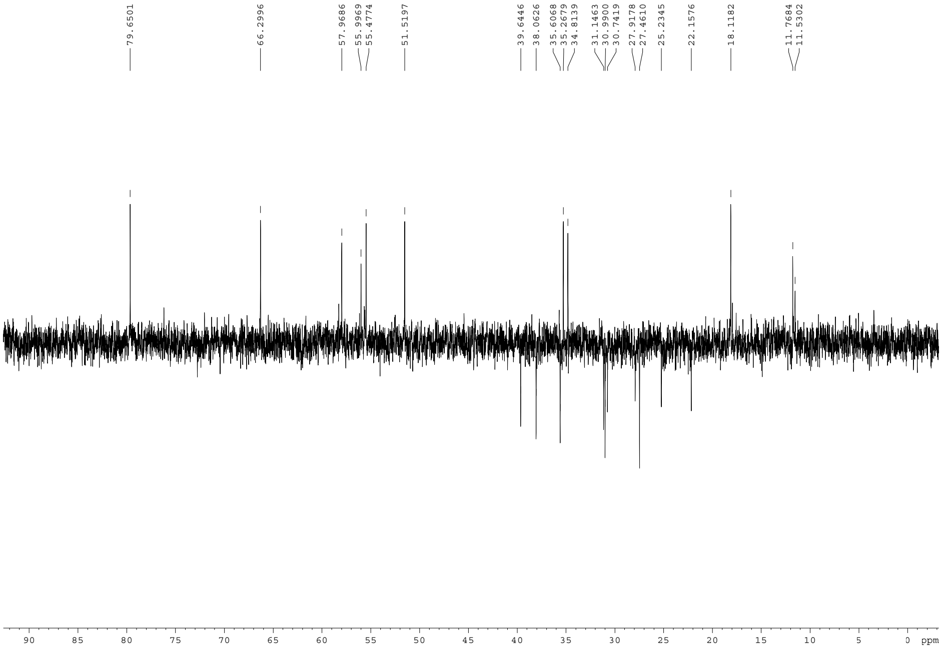

Figure S47. ^{13}C DEPT-135 NMR of Methyl 3α-hydroxy-6-oxa-7-oxo-5α-cholan-24-oate (**16**).

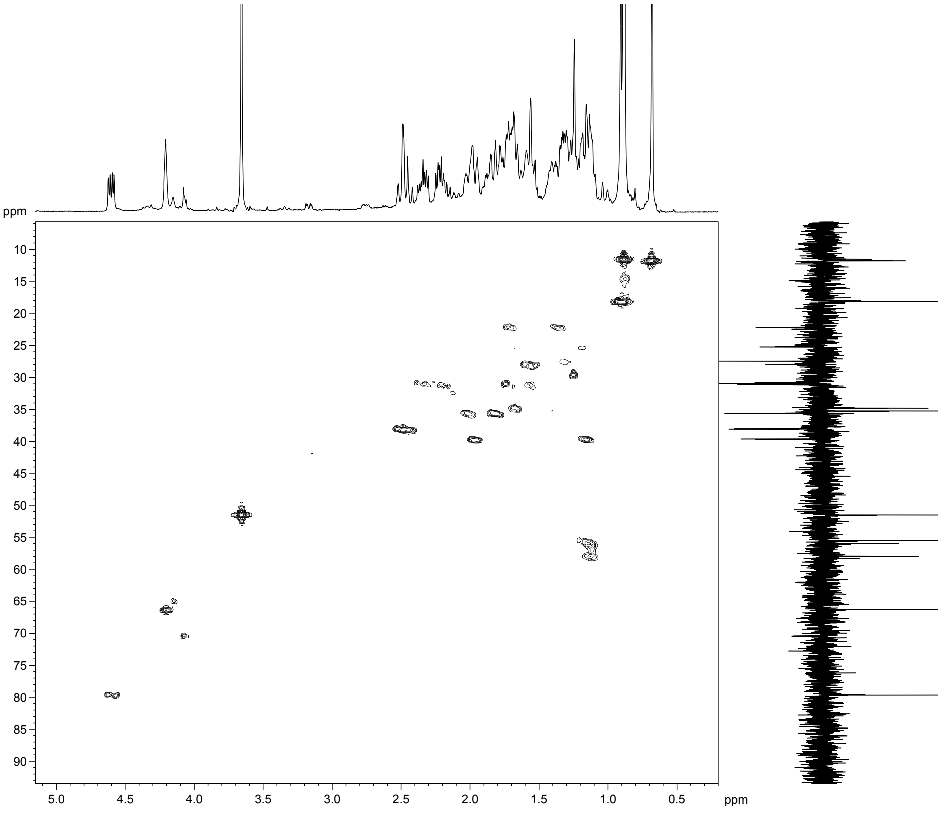

Figure S48. ^1H-^{13}C HSQC of Methyl 3α-hydroxy-6-oxa-7-oxo-5α-cholan-24-oate (**16**).

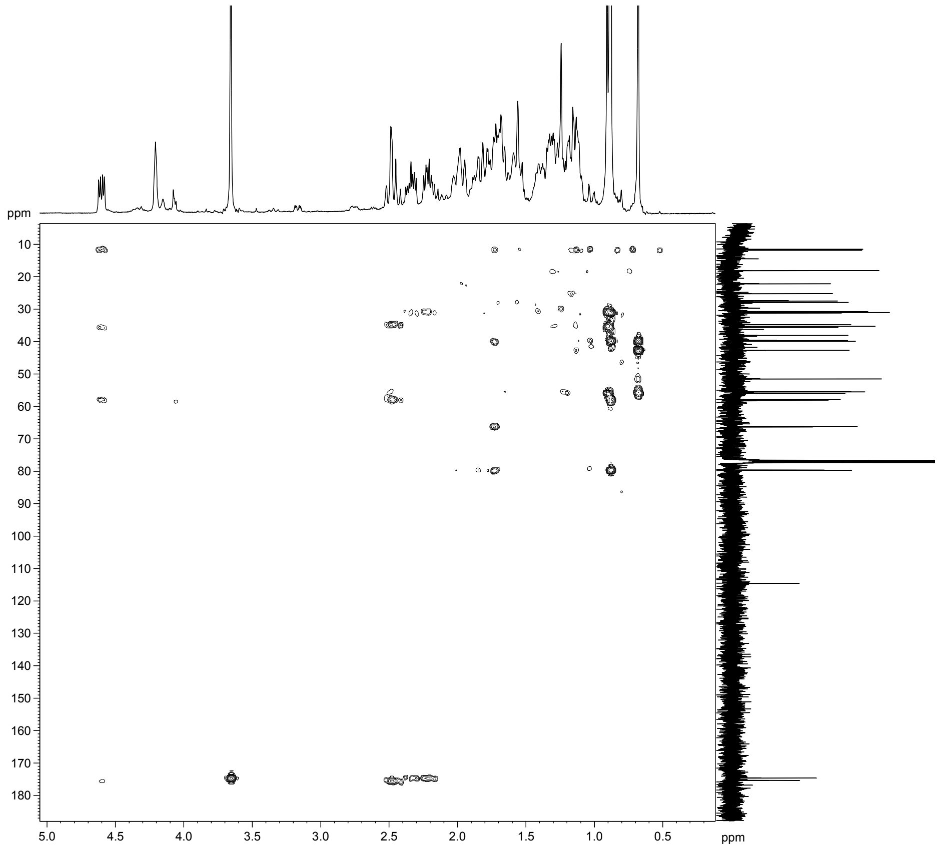

Figure S49. ^1H-^{13}C 2D HMBC of Methyl 3α-hydroxy-6-oxa-7-oxo-5α-cholan-24-oate (**16**).
